# Amyloid-β accumulation in the CNS in human growth hormone recipients in the UK

**DOI:** 10.1007/s00401-017-1703-0

**Published:** 2017-03-27

**Authors:** Diane L. Ritchie, Peter Adlard, Alexander H. Peden, Suzanne Lowrie, Margaret Le Grice, Kimberley Burns, Rosemary J. Jackson, Helen Yull, Michael J. Keogh, Wei Wei, Patrick F. Chinnery, Mark W. Head, James W. Ironside

**Affiliations:** 1 National CJD Research & Surveillance Unit, Centre for Clinical Brain Sciences, Deanery of Clinical Medicine, University of Edinburgh, Edinburgh, EH4 2XU UK; 2 UCL Great Ormond Street Institute of Child Health, 30 Guilford Street, London, WC1N 1EH UK; 3 Centre for Cognitive and Neural Systems, University of Edinburgh, 1 George Square, Edinburgh, EH8 9JZ UK; 4 Department of Clinical Neurosciences, University of Cambridge, Cambridge Biomedical Campus, Cambridge, CB2 0QQ UK; 5 MRC Mitochondrial Biology Unit, University of Cambridge, Cambridge Biomedical Campus, Cambridge, CB2 0XY UK

**Keywords:** Amyloid β, Iatrogenic Creutzfeldt–Jakob disease, Human growth hormone, Prion protein, Cerebral amyloid angiopathy, Neuropathology

## Abstract

Human-to-human transmission of Creutzfeldt–Jakob disease (CJD) has occurred through medical procedures resulting in iatrogenic CJD (iCJD). One of the commonest causes of iCJD was the use of human pituitary-derived growth hormone (hGH) to treat primary or secondary growth hormone deficiency. As part of a comprehensive tissue-based analysis of the largest cohort yet collected (35 cases) of UK hGH-iCJD cases, we describe the clinicopathological phenotype of hGH-iCJD in the UK. In the 33/35 hGH-iCJD cases with sufficient paraffin-embedded tissue for full pathological examination, we report the accumulation of the amyloid beta (Aβ) protein associated with Alzheimer’s disease (AD) in the brains and cerebral blood vessels in 18/33 hGH-iCJD patients and for the first time in 5/12 hGH recipients who died from causes other than CJD. Aβ accumulation was markedly less prevalent in age-matched patients who died from sporadic CJD and variant CJD. These results are consistent with the hypothesis that Aβ, which can accumulate in the pituitary gland, was present in the inoculated hGH preparations and had a seeding effect in the brains of around 50% of all hGH recipients, producing an AD-like neuropathology and cerebral amyloid angiopathy (CAA), regardless of whether CJD neuropathology had occurred. These findings indicate that Aβ seeding can occur independently and in the absence of the abnormal prion protein in the human brain. Our findings provide further evidence for the prion-like seeding properties of Aβ and give insights into the possibility of iatrogenic transmission of AD and CAA.

**Electronic supplementary material:**

The online version of this article (doi:10.1007/s00401-017-1703-0) contains supplementary material, which is available to authorized users.

## Introduction

Prion diseases are transmissible neurodegenerative disorders characterised by the accumulation of a disease-associated misfolded form of the normal cellular prion protein (PrP^C^) in the central nervous system (CNS), commonly referred to as PrP^Sc^ [24]. PrP^Sc^ is considered to be the major, if not the sole, component of the transmissible agents known as prions [55]. Unlike other neurodegenerative diseases, human prion diseases occur in sporadic, genetic and acquired forms [24]. The acquired forms of human prion disease include kuru, variant Creutzfeldt–Jakob disease (vCJD) and iatrogenic Creutzfeldt–Jakob disease (iCJD). One of the commonest causes of iCJD was treatment with human pituitary-derived growth hormone (hGH) by intramuscular or subcutaneous injection in children and young adults with primary or secondary growth hormone deficiency [8, 64]. Treatment with hGH was first associated with iCJD in 1985, since when the use of hGH was banned in many countries and replacement therapy with biosynthetic growth hormone was instigated [19, 34, 54]. Since 1985, over 240 cases of iCJD in hGH recipients have been reported in several countries [8, 50], with the largest numbers of cases occurring in France (119 cases) and the United Kingdom (UK) (78 cases). Although the last case of hGH-iCJD in France occurred in 2009 [4], deaths from hGH-iCJD continue to occur in the UK, with the most recent death occurring in 2016, over 30 years since hGH therapy was banned in the UK [57, 59].

Other more common neurodegenerative disorders are also characterised by the accumulation of abnormal proteins in the CNS, such as amyloid beta (Aβ) and phospho-tau in Alzheimer’s disease, α-synuclein in Parkinson’s disease and TDP-43 in frontotemporal dementia [21]. Unlike human prion diseases, there is no current evidence to suggest that individual cases of these neurodegenerative diseases are acquired [11, 41], but there is an increasing body of experimental evidence to indicate that the abnormal protein aggregates in these diseases exhibit prion-like properties and can spread through the CNS in a highly predictable fashion along well-defined neuroanatomical pathways [12, 21, 30, 56, 61, 62]. The term “propagon” has recently been proposed for all misfolded multimeric proteins that can catalyse the misfolding and aggregation of homotypic proteins and lead to the spread of pathological misfolding at molecular, tissue, systemic and infectious levels [13], with only prions (PrP^Sc^) acting as infectious propagons [35]. However, recent reports of Aβ accumulation in the CNS in small numbers of patients with hGH-iCJD and human dura mater (hDM) graft-related iCJD have suggested that Aβ may also have been transmitted iatrogenically in these patients [16, 22, 29]. Both the pituitary gland in some patients with Alzheimer’s disease and dura mater in elderly individuals can contain aggregates of Aβ [28, 36]. There is also evidence of phospho-tau and α-synuclein accumulation in the pituitary gland in aging; α-synuclein can also be detected in the pituitary gland in Parkinson’s disease and Lewy body dementia [23, 25]. However, the claim for Aβ transmission in 4/8 UK hGH-iCJD cases has been questioned in view of the possibility of underlying CNS disorders in these patients that might predispose to Aβ accumulation, casting “seeds of neuroendocrine doubt” [15]. Furthermore, Aβ has been reported to co-localise with PrP amyloid deposits in the CNS in patients with sCJD and various forms of inherited human prion diseases [9, 27, 44, 49], raising further doubts about the significance of Aβ deposition in hGH-iCJD.

In order to address these questions, we have undertaken a detailed neuropathological, biochemical and genetic study of the largest cohort of UK hGH-iCJD cases yet reported, comprising 35 cases. The full biochemical and prion protein genetic data on a subset of 21 cases have been reported separately, along with some preliminary neuropathological findings [57]. Here, we report the detailed pathological findings in both the CNS and a range of non-CNS tissues in relation to prion protein pathology, along with investigations on Aβ, phospho-tau, α-synuclein and TDP-43 accumulation in the CNS. The findings in this cohort are compared to age-matched UK cases of sCJD and vCJD, and in a small group of UK hDM-iCJD cases. Most importantly, we have a control cohort of 12 UK hGH recipients who did not develop iCJD, but died from complications of the underlying medical conditions that caused their hGH deficiency; no similar cohorts have yet been studied in this way. The hGH control group allows the potential influence of iCJD pathology on CNS Aβ accumulation in hGH recipients to be assessed more fully than hitherto possible. The extensive laboratory-based data on these groups of patients is matched by detailed clinical data, including the dates and duration of hGH treatment in the 35 hGH-iCJD cases and the 12 hGH control cases, and the type of hGH preparations that were used during the period of treatment. Genetic data are also available for most cases, including the *APOE* genotype and data on a range of other genes that might influence Aβ accumulation in the CNS of these individuals.

## Materials and methods

### Cases, inclusion criteria and tissue specimens

**Fig. 1:**
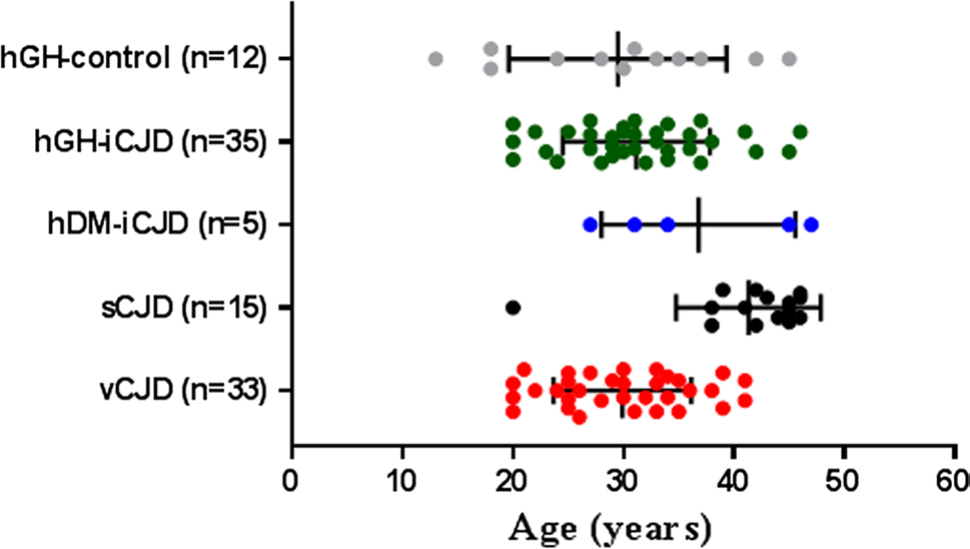
Age distributions of hGH-iCJD and control cases. The sCJD and vCJD control patients were chosen to be as close as possible in range to the hGH-iCJD cases. Cases of sCJD under the age of 50 years are rare; the sCJD control cases are clustered in the 40–50 years age range with higher mean age values. In contrast, cases of vCJD over the age of 40 years are rare; the upper age limit for the vCJD cases is 41 years. *Vertical bars* represent the mean with standard deviation values

All cases included in this study were of UK origin and were referred to the National CJD Research & Surveillance Unit (NCJDRSU) between 1991 and 2016 for neuropathological diagnosis and surveillance purposes. Diagnoses were made according to internationally accepted criteria for human prion diseases [66]. Inclusion criteria for the cases investigated in this study were: a definite diagnosis of hDM-iCJD, hGH-iCJD, or a documented history of treatment with UK hGH with no clinical or neuropathological evidence of a human prion disease (hGH control cases), the availability of formalin-fixed CNS tissue taken at post-mortem and appropriate consent and ethical approval for retention and research use. The study identification number and basic patient data for five hDM-iCJD, 35 hGH-iCJD and 12 hGH control patients who fulfilled these inclusion criteria are detailed in Online Resource Table 1. None of the 35 hGH-iCJD examined in this study were included in the small series of recent UK hGH-iCJD cases reported by Jaunmuktane et al. [29]. The age range at death for the hDM-iCJD (27–47 years), hGH-iCJD (20–46 years) and hGH control cases (13–45 years) are shown in Fig. 1. Thirty-three UK cases of vCJD and 15 sCJD cases of known *PRNP* codon 129 genotype, with age at death ranging from 20 to 46 years, were included as age-matched controls (Fig. 1). All CJD and control tissues were provided by the MRC Edinburgh Brain Bank.

**Table 1:** ABC scores for Alzheimer pathology and CAA scores for Aβ-positive hGH-iCJD, hGH control, sCJD and vCJD cases

Study ID	ABC score (Hyman et al. [26])			Hybrid protocol (from Love et al. [39])			
	A	B	C	Parenchymal CAA	Meningeal CAA	Capillary CAA	Vasculopathy
hGH-iCJD 4	1	0	0	0	0	0	0
hGH-iCJD 5	0	0	0	0	1	0	0
hGH-iCJD 6	0	0	0	1	2	0	0
hGH-iCJD 8	0	0	0	2	2	0	0
hGH-iCJD 9	0	0	0	1	2	0	0
hGH-iCJD 10	1	0	1	2	2	0	0
hGH-iCJD 12	1	0	0	0	0	0	0
hGH-iCJD 15	1	0	0	2	2	0	0
hGH-iCJD 16	1	0	2	1	2	0	0
hGH-iCJD 18	1^a^	0	2	2	2	1	0
hGH-iCJD 19	1	0	0	2	2	0	0
hGH-iCJD 26	0	0	0	0	1	0	0
hGH-iCJD 29	0	0	0	2	2	0	0
hGH-iCJD 31	1	0	1	1	2	0	0
hGH-iCJD 32	1	0	1	2	3	0	0
hGH-iCJD 33	1	0	0	2	2	0	0
hGH-iCJD 34	1	0	0	0	0	0	0
hGH-iCJD 35	1^a^	0	0	0	0	0	0
hGH-control6	1^a^	0	0	0	0	0	0
hGH-control7	1	0	0	0	0	0	0
hGH-control9	0	0	0	2	2	0	0
hGH-control10	1	0	0	0	0	0	0
hGH-control11	1^a^	0	2	3	3	1	1
sCJD14	0	0	0	1	2	0	0
vCJD22	1	0	0	0	0	0	0
vCJD32	0	0	0	0	0	0	0

^a^Cases with diffuse Aβ plaques in the anterior cingulate gyrus in addition to the neocortex, but the full Thal phase 2 distribution of Aβ deposits was absent, e.g. in the entorhinal cortex and hippocampus. These were therefore recorded as Thal phase 1* as recently reported in hDM-iCJD cases [36]

Formalin-fixed, formic acid treated, paraffin-embedded tissue samples from multiple CNS regions (frontal, parietal, occipital and temporal cortices, hippocampus, amygdala, basal ganglia, thalamus, brain stem, cerebellum and spinal cord), including the brain regions recommended in the National Institute on Aging-Alzheimer’s Association guidelines for the assessment of Alzheimer’s disease, were examined whenever possible [26]. In addition, formalin-fixed non-CNS tissue samples were available in 19 of the 35 hGH-iCJD and two of the 12 hGH control cases for examination. All CNS and non-CNS tissue sections were cut at 5 µm for immunohistochemistry and stained for haematoxylin and eosin (H&E). Additionally, sections of the frontal, parietal, occipital and temporal cortices were cut at 8 µm for the Bielschowsky silver stain for neuritic plaques following the protocol described in Dawson et al. [10].

### Antibodies and immunohistochemical analysis

Sections from all CNS tissues were labelled with two monoclonal anti-PrP antibodies recognising different epitopes of the prion protein: 12F10 (amino acids 142–160, Bioquote Ltd, UK) and KG9 (amino acids 140–180, TSE Resource Centre, Roslin Institute, UK). CNS tissue sections were stained by immunohistochemistry for Aβ (6F3D, Dako, UK and 4G8, BioLegend, UK), Aβ 1–40 (BioLegend, UK), Aβ 1–42 (BioLegend, UK), phospho-tau (Thermo Scientific, UK), TDP-43 (2BScientific, UK), and α-synuclein (AJ Roboscreen GmbH, Germany). CNS blocks from selected hGH-iCJD and hGH control cases were also labelled with apolipoprotein E (pan-apoE, BioLegend, UK), apoE-4 (BioLegend, UK), GFAP (Dako, UK), ubiquitin (Dako, UK) and CD68 (Dako, UK). Non-CNS tissues were labelled with the anti-PrP (12F10 and KG9) and anti-Aβ antibodies (6F3D and 4G8). Immunohistochemistry was carried out using the sensitive Novolink™ Polymer Detection System (Leica Biosystems, UK). Details of all antibodies used, including pretreatment protocols, dilutions, incubation times are presented in Online Resource Table 3. All washes were carried out in Tris-buffered saline (TBS) (50 mM Tris; 150 mM NaCl; pH 7.6) with primary antibodies diluted in antibody diluent (Leica Biosystems, UK). Staining was visualised with 3,3′-diaminobenzidine (DAB).

To investigate co-localisation within the brain, selected CNS sections were double immunostained for Aβ and either PrP or phospho-tau, CD68 or GFAP. The pretreatment protocol required for the 12F10 anti-PrP/6F3D anti-Aβ antibody, PGM1 anti-CD68/6F3D anti-Aβ antibody, AT8 anti-tau/6F3D anti-Aβ antibody or GFAP anti-GFAP/6F3D anti-Aβ antibody was performed as detailed in Online Resource Table 3. Immunolabelling for PrP, CD68, phospho-tau and GFAP was performed first. Labelling of PrP, CD68 and phospho-tau was carried out in combination with the Vectastain Elite ABC HRP (Peroxidase, Mouse IgG) Kit (Vector Laboratories, UK; Product PK-6102). Labelling for GFAP was performed in combination with the Vectastain Elite ABC HRP (Peroxidase, Rabbit IgG) Kit (Vector Laboratories, UK; Product PK-6101). Incubations in the primary antibody; 12F10 (1/800), PGM1 (1/50), AT8 (1/100) and GFAP (1/800) were carried for 1 h and staining visualised with 3,3′-diaminobenzidine (DAB). Staining was continued for Aβ protein with sections blocked in normal rabbit serum for 20 min before incubating in the primary antibody 6F3D (1/250) overnight at room temperature. Sections were incubated in a biotin-SP-conjugated affinity pure rabbit anti-mouse IgG antibody (Jackson Immunoresearch Laboratories Inc, USA; Product 315-065-003) at a dilution of 1/400 in TBS for 1 h before a final incubation in Vectastain ABC alkaline phosphatase standard (Vector Laboratories, UK; Product AK-5000) for 30 min. Staining was visualised with Vector red alkaline phosphatase substrate kit (Vector Laboratories, UK, catalogue PK-SK-5100).

### Assessment of CNS tissues for prion-related pathology

Tissue sections were analysed independently by two experienced assessors (DLR and JWI). H&E sections from all CNS regions were assessed for the distribution, severity and nature of spongiform change, neuronal loss, gliosis and amyloid plaque formation and scored in a semiquantitative manner [46] using 4 scores (0—absent, 1—mild, 2—moderate, 3—severe). All CNS sections from the hGH-iCJD cases stained with the anti-PrP antibodies were examined to determine the distribution, severity and nature of the abnormal PrP accumulation (granular, perineuronal/pericellular, plaque-like, plaques, amorphous deposits, perivascular, vascular). The findings were assessed in conjunction with those from the examination of the H&E sections to allow subclassification by histotyping [46, 47] to determine whether the patterns of neuropathology observed in the hGH-iCJD cases resembled those occurring in recognised sCJD subtypes or not.

### Assessment of Aβ and phospho-tau pathology

An “ABC” score for the level of Alzheimer’s disease neuropathology was calculated for each hGH-iCJD, hGH control, hDM-iCJD, vCJD control and sCJD control case according to Hyman et al. [26]. This score was derived from the classification of the Aβ phase on sections stained using the Aβ immunohistochemistry (“A” score: Thal phase for Aβ deposits) [65], the Braak and Braak neurofibrillary change stage using immunohistochemistry for phospho-tau (“B” score: Braak & Braak stage for neurofibrillary changes) [5, 6] and the neuritic plaque score on Bielschowsky silver-stained sections by the method of the Consortium to Establish a Registry for Alzheimer’s Disease (CERAD) (“C” score: the CERAD score for neuritic plaques) [43]. All cases were also assessed for cerebral amyloid angiopathy (CAA) in vessels in the leptomeninges, the brain parenchyma and brain capillaries. CAA was scored according to the criteria of Love et al. [39]. In addition, cases with CAA were also stained with the anti-Aβ 1–40 antibody and the anti-Aβ 1–42 antibody and the distribution of immunoreactivity for each antibody in the CAA blood vessels was recorded.

### Immunohistochemistry for apoE and apoE-4

Sections of the CNS from cases of hGH-iCJD and hGH controls for which no frozen tissue was available for *APOE* genotype analysis were stained with antibodies to apoE and apoE-4 (Online Resource Table 2). As in a previous publication using apoE immunohistochemistry [60], the specificity of immunolabelling with these antibodies was confirmed on cases of Alzheimer’s disease of known *APOE* genotypes (Ɛ3/3, Ɛ3/4 and Ɛ4/4) as positive controls for apoE and apoE-4 expression, with appropriate negative controls. The hGH-iCJD cases and hGH control cases with CNS Aβ accumulation that showed selective labelling with both anti-apoE-4 antibodies were judged to be cases with an apoE-4 phenotype and categorised as apoE-4 +ve (IHC); cases with CNS Aβ accumulation but no evidence of apoE-4 labelling were categorised as apoE-4-ve (IHC).

**Table 2:** *APOE* genotypes in hGH-iCJD and hGHcontrol cases

*APOE* genotype and apoE-4 phenotype	hGH-iCJD		hGH control	
	CNS Aβ accumulation		CNS Aβ accumulation	
	Positive	Negative^a^	Positive	Negative^a^
2/2	0	0	0	0
2/3	1	1	0	0
2/4	0	0	0	0
3/3	8	6	1	0
3/4	4	2	1	0
4/4	0	0	0	0
ApoE4 +ve^b^ (IHC)^a^	0	0	1	0
ApoE4 −ve^b^ (IHC)^a^	5	0	2	0
Total	18	9	5	0

^a^No information on the *APOE* genotype or ApoE-4 phenotype can be obtained on the remaining 6 hGH-iCJD cases and the 7 hGH control cases with no CNS Aβ accumulation, due to a lack of frozen tissue samples and the absence of Aβ pathology

^b^apoE-4 phenotypes were determined on paraffin sections of the brains from 5 hGH-iCJD and 3 hGH-control cases with CNS Aβ accumulation using immunohistochemistry for the apoE-4 protein

### Paraffin-embedded tissue blot

Paraffin-embedded tissue (PET) blotting was performed on all formalin-fixed, non-CNS tissues from hGH-iCJD and hGH control cases as previously described [58]. PET blotting was performed using two monoclonal anti-PrP antibodies recognising different epitopes of the prion protein; 3F4 (amino acids 109–112, Cambridge Bioscience, UK) and 12F10 in combination with the Vectastain ABC-AmP kit (Vector Laboratories, Peterborough, UK; Product AK-6400). Briefly, 5-µm tissue sections were mounted onto 0.45-µm nitrocellulose membrane and incubated overnight at 55 °C. Nitrocellulose-mounted tissue sections were deparaffinised before an overnight digestion in 25 µg/ml proteinase K (Roche Diagnostics; Product 03115 836 001). Membranes were washed in TBST (10 mM Tris HCl pH 7.8, 100 mM NaCl, 0.05% Tween 20) before treatment with 3 mol/l guanidine isothiocyanate for 10 min. After a further wash tissue sections were blocked with casein and incubated for 2 h in the primary antibodies (12F10, 1/22 000; 3F4, 1/500) diluted in casein. Immunolabelling was completed using the Vectastain ABC-AmP kit and staining visualised using the nitro blue tetrazolium/5-bromo-4-chloro-3-indolyl phosphate (NBT/BCIP) chromogen system.

### APOE genotyping

*APOE* genotyping was performed by competitive allele-specific PCR, using KASP™ genotyping assays (LGC Genomics, Hoddesdon, UK) for both rs7412 and rs429358. Subsequent genotype data was converted into *APOE* allele status [70]. Full methods for the KASP™ genotyping platform are available from LGC Genomics (http://www.lgcgenomics.com/genotyping/kasp-genotyping-reagents/).

### Exome sequencing and analysis

As previously described [31], genomic DNA was fragmented, exome enriched and sequenced (Nextera Rapid Exome Capture 62 Mb or TruSeq rapid 37 Mb kit on a HiSeq 2000 with 100 bp paired-end reads). Bioinformatic analysis was performed using an in-house pipeline including alignment (human reference genome hg19, UCSC) using Burrows-Wheeler Aligner (BWA) [38]. Variant calling was performed using FreeBayes [18]. Subsequent analysis was restricted to on-target homozygous, heterozygous, and compound heterozygous variants with a minimum read depth of 10, and base quality score of 20. Further analysis selected variants in 5′ untranslated region (UTR), 3′ UTR or exonic regions within genes of interest that may influence the aggregation of Aβ in the CNS (APP, C9ORF72, CHMP2B, CSFIR, FUS, GRN, ITM2B, MAPT, NOTCH3, PSEN1, PSEN2, SERPINI1, SQSTM1, TARDBP, TREM2, TYROBP, VCP) and with a minor allele frequency <5% in the 1000 Genome Project Database [1] of European/American cases from the NHLBI ESP exomes database [45] and the ExAC server [14], using Qiagen Ingenuity Variant Analysis software (Qiagen, Hilden, Germany).

### Western blotting with sodium phosphotungstic acid precipitation

Western blot analysis of protease-resistant prion protein (PrP^res^) in non-CNS hGH-iCJD tissues was performed using the highly sensitive sodium phosphotungstic acid (NaPTA) precipitation method as previously described [20, 51, 69]. Briefly, 10% w/v extracts were made by homogenising tissues in an appropriate volume of 2% sarkosyl/phosphate-buffered saline (PBS), pH 7.4, using the FastPrep instrument (Qbiogene, Cambridge, UK). The samples were then cleared by centrifugation at 5200*g* for 5 min at 4 °C. Cleared spleen tissue homogenate (5%) from a non-CJD patient was used as a negative control. In addition, iCJD or sCJD brain tissue homogenate (10%, 10 µl corresponding to 100 µg brain) was diluted and mixed into 1 ml of 5% cleared non-CJD spleen tissue homogenate, for use as a positive control. Samples (0.5 ml) of the cleared lysates were diluted with a further 0.5 ml of 2% sarkosyl/PBS and incubated for 10 min at 37 °C. Benzonase (Sigma, UK) and MgCl_2_ were added at final concentrations of 50 U/ml and 1 mmol/l, respectively, and incubation at 37 °C was continued for a further 30 min. 81 μl of a stock solution of 4% w/v NaPTA and 170 mmol/l MgCl_2_, pH 7.4, were added (final concentration of NaPTA, 0.3% w/v), and precipitation developed for 30 min at 37 °C. Samples were centrifuged at 20,800*g* for 30 min at 37 °C. The resultant supernatant was discarded and the pellets resuspended in 20 μl of 0.1% w/v sarkosyl in PBS, pH 7.4, and digested with 50 μg/ml proteinase K for 30 min. Digestion was terminated by the addition of 1 mmol/l PefaBloc SC (Roche, UK). Electrophoresis was performed using the NuPAGE Novex gel system (Invitrogen). Before electrophoresis, NuPAGE LDS sample buffer was added to each of the samples to a final concentration of 1×. Samples were boiled for 10 min and separated on 10% Bis–Tris NuPAGE gels. The separated proteins were then transferred onto PVDF membrane (Bio-Rad, UK). For immunodetection, the anti-PrP monoclonal antibody 3F4 (Dako, UK) was used at a final concentration of 50 ng/ml IgG for 1 h. Horseradish peroxidase-conjugated anti-mouse IgG F(ab′)_2_ fragment (Sigma-Aldrich, Dorset, UK)) was used at a dilution of 1 in 40,000 for 1 h. The detection reagent used was SuperSignal West Femto Maximum Sensitivity Substrate (Thermofisher Scientific).

### Statistical analysis

Advice on appropriate methods for statistical analyses was kindly provided by Dr Anna Molesworth, Senior Epidemiologist, NCJDRSU, University of Edinburgh and Catriona Graham, Lead Statistician, Edinburgh Clinical Research Facility, University of Edinburgh.

Excel (Microsoft, Reading, UK) and Prism 7.00 (GraphPad Software, Inc, La Jolla, USA, on license) were used for data storage and processing for statistical analysis of the data. The threshold used for statistical significance was *p* < 0.05. All graphs were generated using GraphPad Prism 7.00 (GraphPad Software, Inc, La Jolla, USA, on license).

## Results

### Clinical features

Thirty-five autopsy cases of hGH-iCJD with formalin-fixed CNS tissue were available for neuropathological analysis. The study identification numbers and clinical data including; sex, age at death, age at disease onset, duration of illness, incubation period, *PRNP* codon 129 genotype, duration of hGH treatment, the mid-point of treatment to death and the original diagnosis for all 35 patients are shown in Online Resource Tables 1 and 2. In addition, clinical summaries of 12 hGH recipients with no clinical or neuropathological evidence of a human prion disease (hGH control cases) were available for examination and are detailed in Online Resource Tables 1 and 2. A full biochemical characterisation and prion protein genetic analysis of 21 of the 35 hGH-iCJD (hGH-iCJD1–hGH-iCJD21) patients in which frozen autopsy tissue was available has been published separately, along with some preliminary neuropathological findings [57]. The age range at death was similar for the hGH-iCJD (20–46 years, mean 31.2 years ± SD 6.7 years) and hGH control (13–45 years, mean 29.5 ± 9.8 years) cases (Fig. 1). *PRNP* codon 129 genotype was available in 30 of these 35 hGH-iCJD cases and confirmed our earlier findings that the MV (15 cases) and VV (11 cases) codon 129 genotypes dominate in UK hGH-iCJD cases and that these tended to occur earlier in the hGH-iCJD epidemic than the MM *PRNP* codon 129 cases (4 cases) [57]. This is in contrast to sCJD in the UK where the MM *PRNP* codon 129 genotype is the most frequently occurring group.

**Fig. 2:**
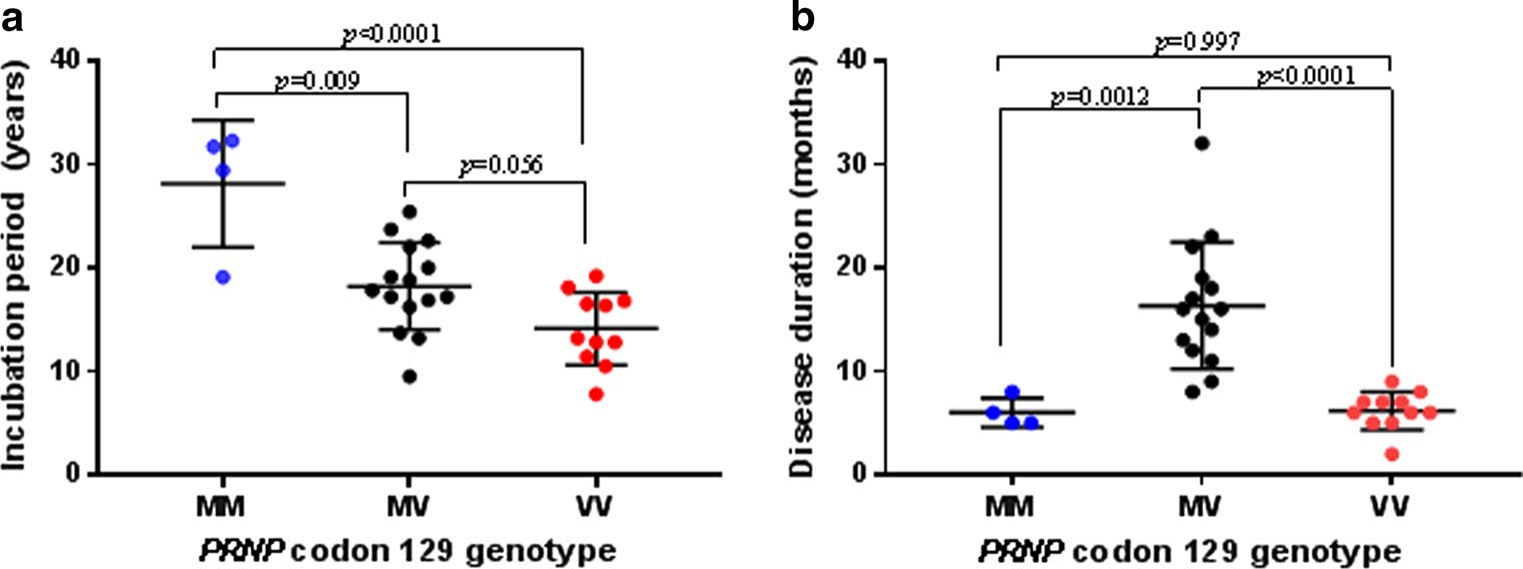
Relationship of *PRNP* codon 129 polymorphisms to hGH-iCJD incubation period and disease duration. **a** Differences in the incubation periods for hGH-iCJD in relation to *PRNP* codon 129 genotype. **b** Differences in the hGH-iCJD disease duration in relation to *PRNP* codon 129 genotype. Statistical analyses were performed using one-way ANOVA followed by Tukey’s multiple comparison test; *horizontal bars* represent mean with standard deviation values

All hGH patients examined had received treatment with hGH during the period 1969–1985 with the duration of hGH treatment varying from 2–11.2 years for the hGH-iCJD cases to 1–12.3 years for the hGH control cases (Online Resource Table 2). One form of UK pituitary-derived hGH, the modified Wilhelmi preparation, had been administered to all hGH recipients who had developed iCJD, albeit in varying quantities and over different time periods [59, 64]. All 35 hGH-iCJD patients in this study had received hGH produced by the modified Wilhelmi method for treatment periods of between 6 months and 8.2 years. In contrast, only eight of the 12 hGH-control patients received the modified Wilhelmi preparation for treatment periods ranging from 4 months to 6.6 years. In agreement with our earlier data, statistically significant associations were found between the *PRNP* codon 129 genotype and disease incubation periods and duration of illness in the hGH-iCJD patients [57]. The four codon 129 MM hGH-iCJD patients had the longest incubation periods in comparison to the MV and the VV patients. In contrast, codon 129 MV patients have a significantly longer duration of illness in comparison to the MM and VV patients (Fig. 2). Clinical data from five hDM-iCJD autopsy cases with formalin-fixed CNS tissue available for neuropathological analysis are also provided in Online Resource Table 1.

### Neuropathological phenotype in hGH-iCJD and hDM-iCJD

The neuropathological features were reviewed in all 35 hGH-iCJD cases. The distribution, severity and nature of the spongiform change, gliosis, amyloid plaque formation and the accumulation of disease-associated prion protein on immunohistochemistry were recorded in all cases (fixed tissue from case hGH-iCJD1 was not available for PrP immunohistochemical analysis). A widespread spongiform encephalopathy was present in all cases, accompanied by variable neuronal loss and gliosis, with amyloid plaques in the cerebellum identified in 16 cases. In general, the distribution and nature of the pathological features in the CNS in the hGH-iCJD cases showed close similarities to those in sCJD cases with the corresponding *PRNP* codon 129 genotype, allowing a histotype to be assigned to each case. The histotype assigned to each hGH-iCJD case is detailed in Online Resource Table 1.

**Fig. 3:**
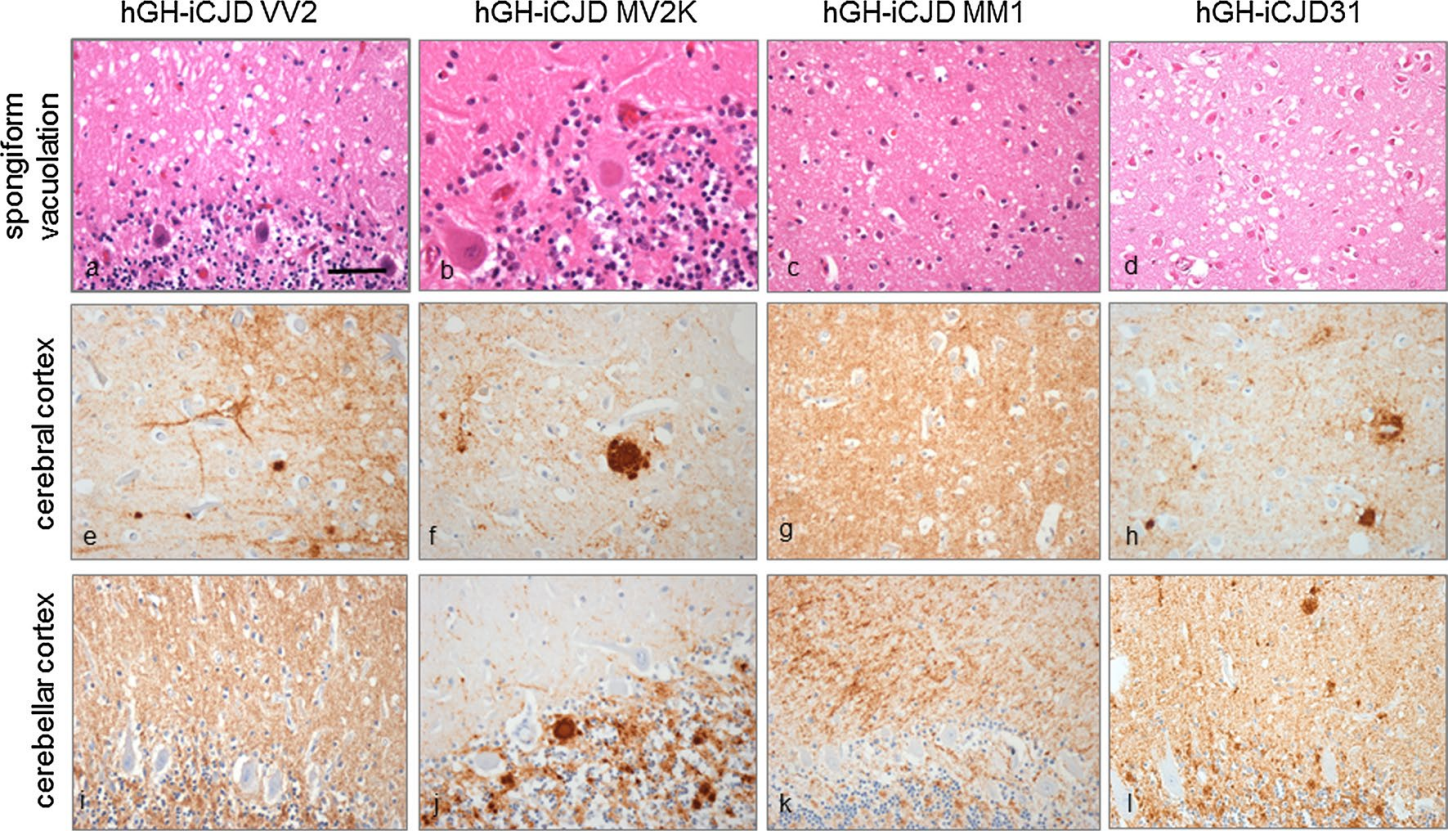
Typical neuropathological phenotypes in UK hGH-iCJD. *PRNP* codon 129 VV hGH-iCJD cases show microvacuolation in the cerebral cortex (**a**) with a combination of granular, perineuronal and plaque-like accumulations of PrP in the cerebral cortex (**e**). The cerebellar cortex in VV hGH-iCJD cases shows a predominantly granular pattern of PrP accumulation (**i**). *PRNP* codon 129 MV hGH-iCJD were characterised by the presence of kuru plaques in the cerebellar and occasionally in the cerebral cortex (**b**, **f**, **j**). *PRNP* codon 129 MM hGH-iCJD cases show widespread microvacuolation in the cerebral cortex (**c**) with a predominantly granular accumulation of PrP in the cerebral (**g**) and cerebellar cortex (**k**). Case hGH-iCJD31 shows atypical neuropathological features in comparison with the codon 129 MM hGH-iCJD cases. Microvacuolation was observed in the lower layers of the cerebral cortex (**d**) with a combination of granular, perineuronal and plaque-like accumulations of PrP in the cerebral and cerebellar cortex (**h**, **l**). No kuru-type amyloid plaques were present in any brain region. These features show some similarities to the VV2 histotype in sCJD. Sections **a**, **c**, **e**, and **g**, are stained with H&E and sections **b**, **d**, **f**, and **h** are stained with the KG9 antibody. The *bar* in **a** represents 25 µm for **a**, **c**–**l**; and 15 µm for **b**

Three major histotypes were identified in relation to the established classification of subtypes of sCJD [47]. All 11 codon 129 VV cases showed a pattern closely resembling that of the sCJD VV2 histotype characterised by microvacuolation, often in a linear distribution in layer 5 of the cerebral cortex with severe spongiform change in the basal ganglia, the CA1 region of the hippocampus and the subiculum. Severe neuronal loss and gliosis was apparent in the cerebellar cortex, often resulting in cerebellar cortical atrophy (Fig. 3a). PrP immunohistochemistry shows granular and perineuronal deposits in the cerebral cortex in layer 5, with decoration of apical ascending dendrites. Plaque-like deposits occurred throughout the brain, but no true amyloid plaques were detected (Fig. 3e, i). Three additional cases (hGH-iCJD29, 30 and 32) for which no *PRNP* codon 129 genotype data were available had neuropathological features corresponding to the sCJD VV2 histotype.

Fourteen of the 15 codon 129 MV patients showed a pathology closely associated with the sCJD MV2K histotype with predominantly microvacuolation in the cerebral cortex, hippocampus, basal ganglia and thalamus and cerebellar cortex. The presence of occasional areas of confluent spongiform change in the cerebral cortex in four cases allowed further subclassification as MV2K + 2C. Kuru-type amyloid plaques were present in the cerebellum in all 14 cases, and occasionally in the cerebral cortex. Perineuronal, kuru-type plaques and plaque-like PrP deposits were observed in the cerebellum, basal ganglia, thalamus, hippocampus and occasionally in the cerebral cortex (Fig. 3b, f, j). The remaining codon 129 MV case had a pathology more in keeping with the sCJD MV2C histotype, with a predominantly confluent spongiform change in the cerebral cortex and a perivacuolar pattern of PrP deposition. Kuru-type amyloid plaques were not observed in this case. One additional case for which no *PRNP* codon 129 genotype could be determined (hGH-iCJD33), had neuropathological features corresponding to the sCJD MV2K histotype.

Two of the four codon 129 MM cases showed features associated with the sCJD MM1/MV1 subtype, with a widespread spongiform encephalopathy of microvacuolar type, most severe in the frontal and occipital cortex. Patchy spongiform change was observed in the cerebellar cortex, with no amyloid plaques. PrP deposition showed a widespread granular pattern (Fig. 3c, g, k). One additional case (hGH-iCJD25) for which no *PRNP* codon 129 genotype data were available had neuropathological features corresponding to the MM1/MV1 histotype. Four of the five hDM-iCJD cases showed a neuropathological phenotype closely resembling that of sCJD MM1/MV1 histotype, while hDM-iCJD4 resembled the sCJD VV2 histotype. Kuru-type amyloid plaques were not observed in any hDM-iCJD cases. No evidence of a spongiform encephalopathy or prion protein immunoreactivity was found in any of the hGH-control cases.

### Atypical neuropathology in hGH-iCJD

Two *PRNP* codon 129 MM cases (hGH-iCJD20 and hGH-iCJD31) showed neuropathological features that did not correspond to those described by Parchi et al. [47] in MM codon 129 cases of sCJD. Case hGH-iCJD20 was reported in detail in our earlier publication [57] and showed the presence of kuru plaques and PrP plaque-like deposits in the cerebellum and cerebral cortex in a phenotype more in keeping with that of sCJD MV2K subtype (sCJD MV2K + 2C histotype) than the MM1/MV1 histotype. Case hGH-iCJD31 shared some of the atypical features seen in hGH-iCJD20, with predominantly microvacuolar spongiform change in the cerebral cortex, particularly in layers 5–6, hippocampus and basal ganglia (Fig. 3d). The thalamus and cerebellum showed less spongiform change, but no kuru-type plaques were identified in the cerebellum. PrP immunohistochemistry showed a combination of granular, perineuronal and plaque-like PrP deposits in a widespread distribution in the CNS. An occasional PrP plaque-like deposit was present in the parietal cortex, with intense labelling on PrP immunohistochemistry (Fig. 3h, l). This phenotype was distinct from both the MM/MV1-like histotype and the MV2-like histotype in hGH-iCJD, and from MM1 and MV2 histotype in sCJD. However, there are some similarities to the VV2 phenotype in both sCJD and hGH-iCJD.

### Co-pathology and Aβ accumulation in hGH-iCJD and hGH controls

**Fig. 4:**
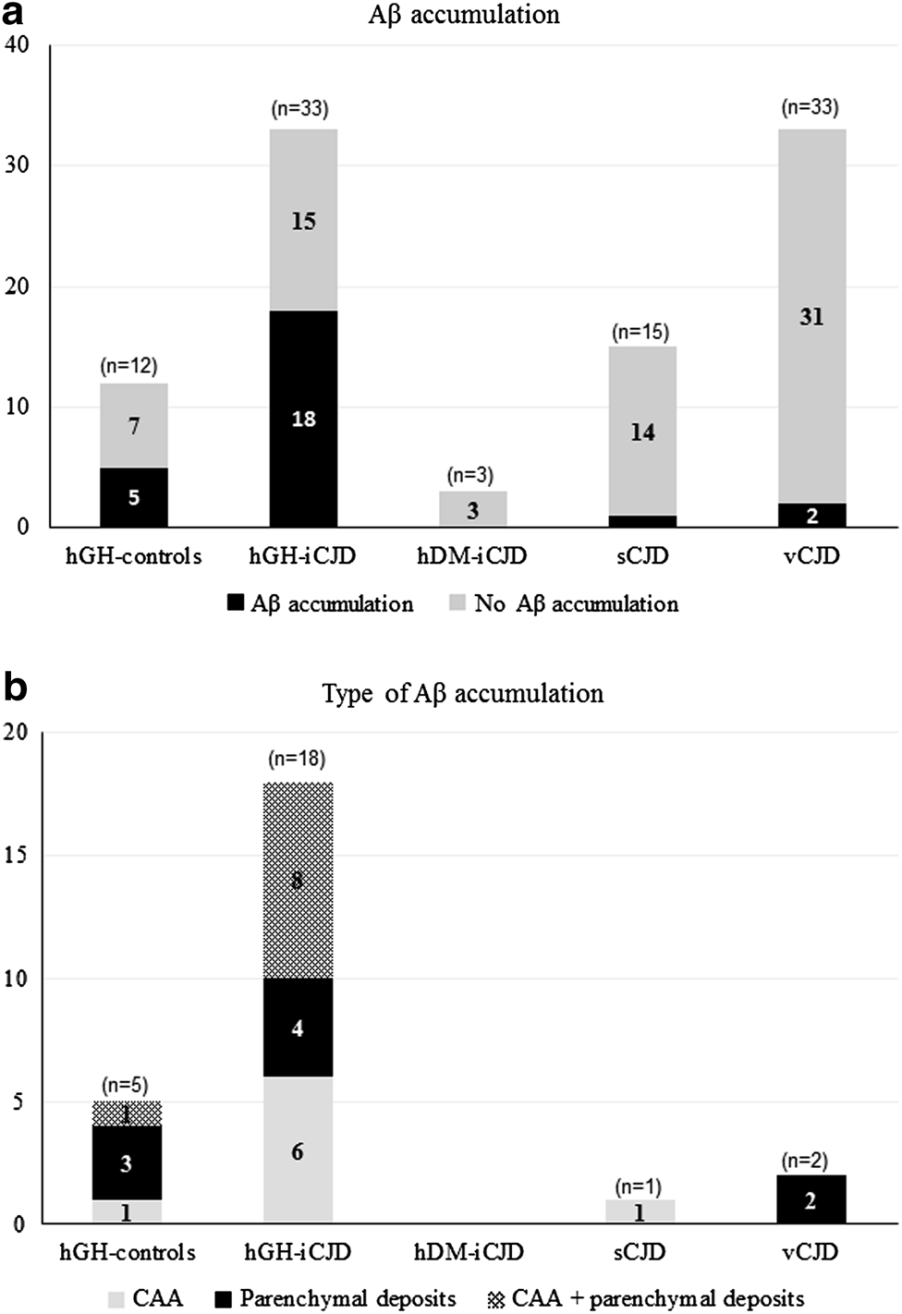
Frequency of Aβ accumulation in hGH-iCJD and control cases. There is a significant difference in the percentage of cases with CNS Aβ deposition in the two groups treated with hGH (51%) compared to the three groups not treated with hGH (6%); *p* < 0.001 (Fisher’s exact test). Comparison of the hGH-iCJD, hGH control and non hGH-treated groups confirms the association between CNS Aβ accumulation and hGH treatment; *p* < 0.001 (Chi-squared test)

**Fig. 5:**
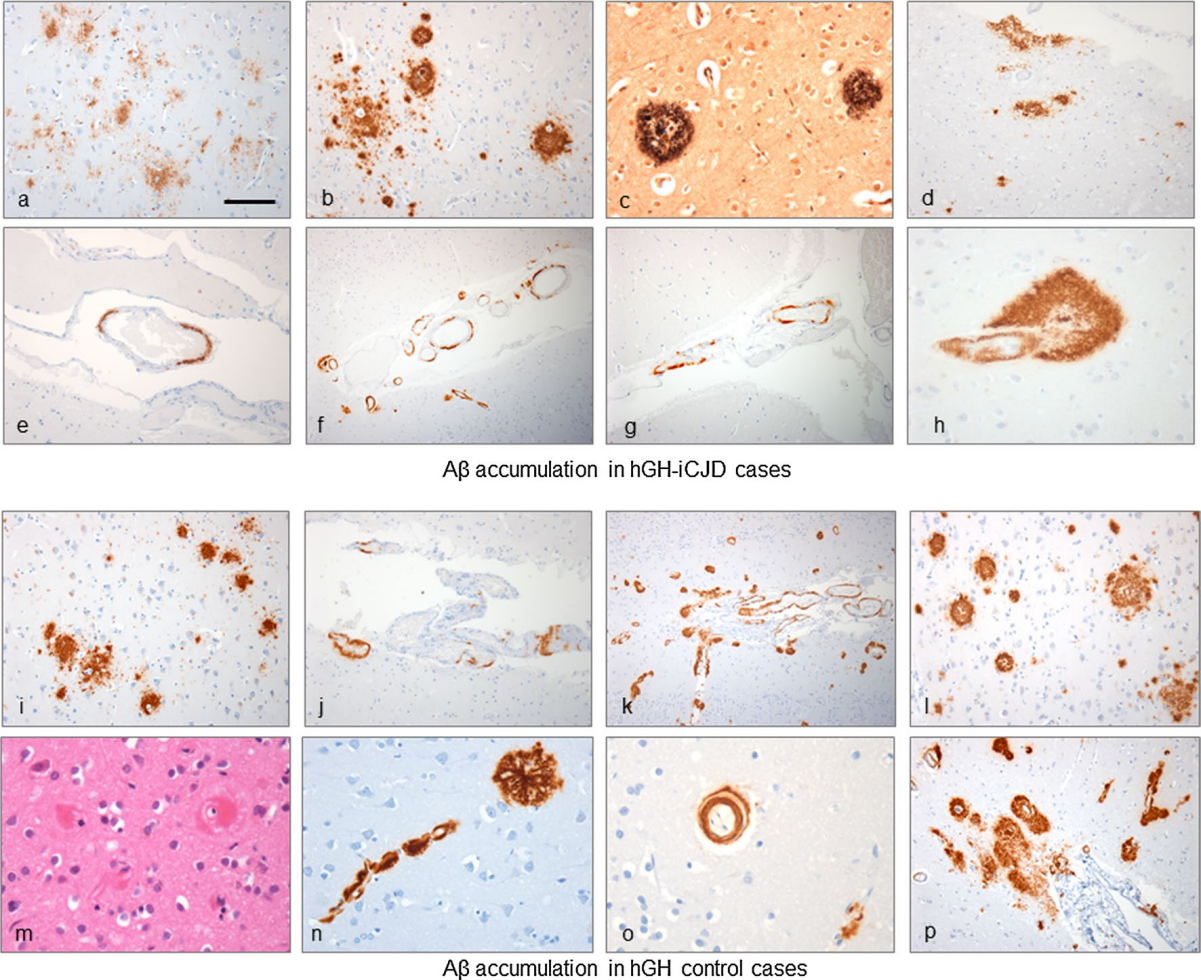
Aβ accumulation in the CNS in hGH-iCJD and hGH control patients. Aβ immunohistochemistry (6F/3D antibody) in hGH-iCJD (**a**–**h**) and hGH control (**i**–**p**) cases. Diffuse plaques were a feature of all hGH-iCJD cases in which CNS Aβ deposition was observed (**a**). Cored plaques were less frequently observed in hGH-iCJD cases (**b**) with neuritic plaques demonstrated with the Bielschowsky silver stain (**c**). Patchy diffuse subpial deposits of Aβ were also a feature of hGH-iCJD cases (**d**). Patchy deposition of Aβ in the wall of a meningeal vessel with a more extensive Aβ deposition in occipital vessels in the meninges and adjacent cortex in hGH-iCJD (**e**, **f**). Patchy Aβ deposition was observed in the wall of meningeal vessels overlying the cerebellar cortex in a single hGH-iCJD case (**g**). Circumferential deposition in a cortical arteriole with extensive perivascular Aβ forming a cored plaque-like structure (**h**). Diffuse Aβ deposits and plaques were also found in the cerebral cortex in hGH control cases (**i**). CAA with patchy meningeal deposits and circumferential deposition were observed in the superficial cortical vessels in hGH control cases (**j**). hGH control11 showed extensive CAA in the meninges and cortex with diffuse and perivascular Aβ deposits and multiple cored plaques and smaller diffuse Aβ deposits (**k**, **l**). This case also showed severe capillary CAA with marked thickening of vessel walls, the presence of dyshoric cortical vessel and vasculopathy shown with the splitting of an intracortical arteriole wall (**m**–**o**). Meningeal and intracortical CAA with diffuse subpial deposits, perivascular deposits and diffuse and cored Aβ plaques in hGH control case11 (**p**). The *bar* in **a** represents 50 µm for **a**, **b**, **d**–**e**, **g**, **i**, **l**, **p**; 20 µm for **h**, **m**–**o**; 25 µm for **c**; and 100 µm for **f**, **j**, **k**

Of the 35 hGH-iCJD cases, 33 had sufficient paraffin-embedded tissue for further pathological analysis by immunohistochemistry. Aβ was detected as CAA and/or CNS parenchymal deposits in 18 of the 33 hGH-iCJD cases. Of these 18 Aβ-positive cases, six had CAA only, four had parenchymal deposits without CAA and eight cases had both CAA and parenchymal deposits (Fig. 4). Parenchymal Aβ deposits occurred in the form of diffuse (immature) and cored plaques, neuritic plaques and subpial deposits in the cerebral cortex in an unpredictable and varied distribution, either singly or in clusters, in one or more cortical regions (Fig. 5a–c). The occurrence of Aβ plaque clusters has also been reported in hDM-iCJD cases, but we found relatively more diffuse Aβ plaques than in the hDM-iCJD cases, which contained predominantly cored Aβ plaques [35, 36]. In the hGH-iCJD cases who had undergone neurosurgery for tumour resection (Online Resource Table 2), we did not observe the accentuation of Aβ accumulation around the operation sites that was reported in hDM-iCJD cases [35, 36]. There was no relationship between the location of the Aβ parenchymal deposits and either the vascular boundary zone areas in the brain or the topography of the cerebral gyri (including the depth of the sulci). Furthermore, we found no relationship between the morphology of the parenchymal Aβ deposits and either the patterns of prion protein accumulation in the brain or the severity or the nature of the spongiform change. No evidence of co-localisation of Aβ deposits with the kuru-type plaques or focal plaque-like deposits in the hGH-iCJD cases was noted. However, it was not possible to establish with certainty that there was clear separation of the Aβ deposits from the widespread granular PrP positivity in affected cortical grey matter in hGH-iCJD cases. All 12 hGH-iCJD cases with parenchymal Aβ deposits contained diffuse plaques, with cored plaques found in 6/12 cases and neuritic plaques identified with the Bielschowsky silver stain in 5/12 cases (Fig. 5c). The plaque frequency varied from occasional diffuse grey mater plaques to more numerous diffuse and neuritic plaques (up to CERAD score 2). Immunohistochemistry for phospho-tau showed only small numbers of fine neuritic processes around the Bielschowsky-positive neuritic plaques. Patchy diffuse subpial Aβ deposits were also present in five cases, but other forms of diffuse Aβ (such as lake-like or fleecy deposits) were absent (Fig. 5d). No Aβ deposits were identified in the entorhinal cortex, hippocampus, basal ganglia, thalamus, brain stem, cerebellum, spinal cord or white matter.

CAA in 14 hGH-iCJD cases varied from occasional focal Aβ deposits in meningeal vessel walls to more extensive circumferential deposition in meningeal and intraparenchymal vessels, with variable perivascular Aβ accumulation (Fig. 5e–h). None of the hGH-iCJD cases showed vasculopathy related to CAA. Case hGH-iCJD18 displayed sparse capillary CAA in addition to parenchymal and meningeal CAA. CAA was present most often in the occipital meningeal vessels and occipital cortex, but occasional cases had isolated CAA in the parietal or frontal cortex. A single case also had CAA in the cerebellar meningeal vessels, with no cerebellar parenchymal involvement (Fig. 5g). None of the CAA-affected vessels were labelled with the anti-PrP antibodies.

**Fig. 6:**
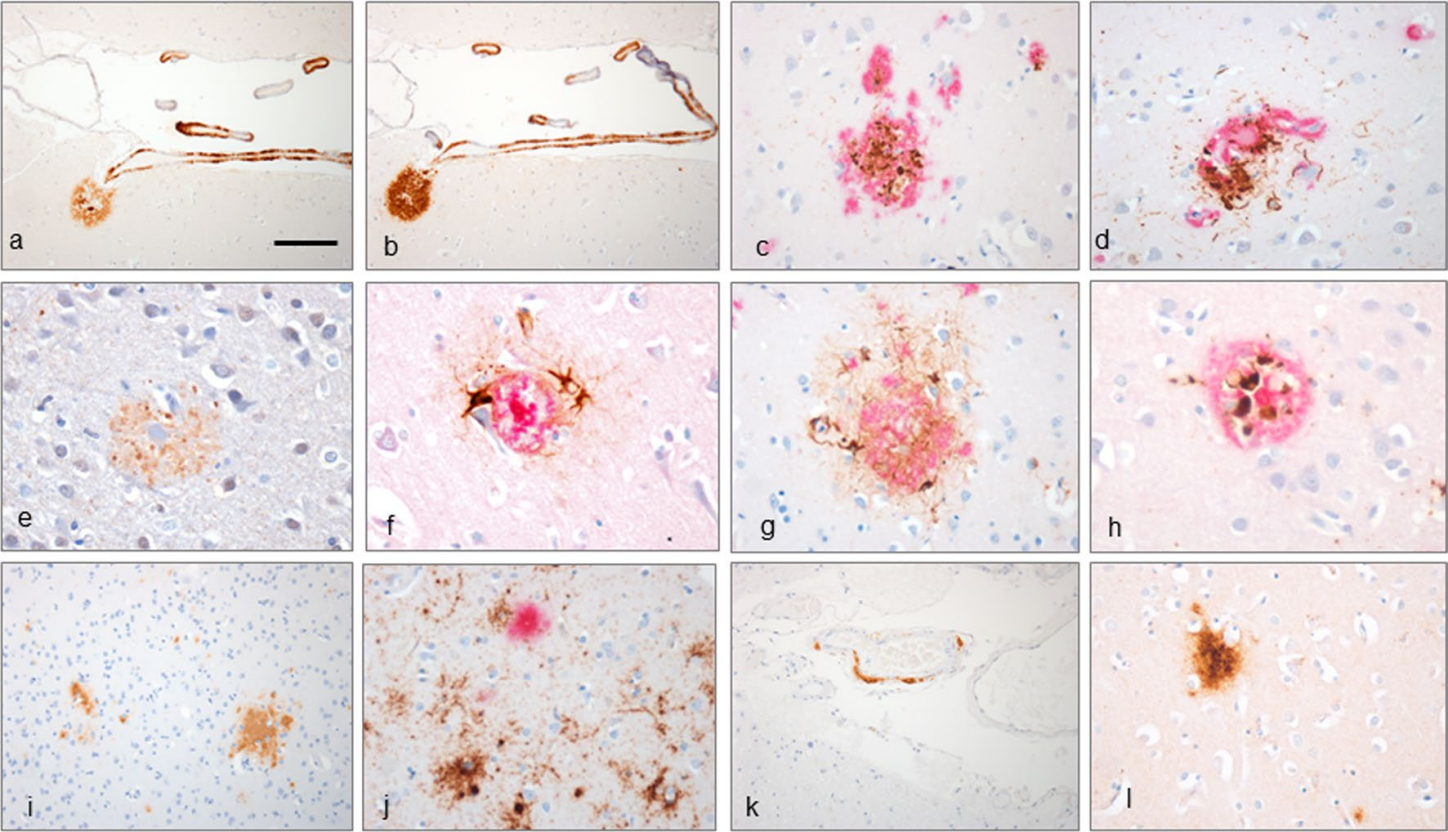
Aβ pathology in hGH recipients, sCJD and vCJD. Immunohistochemistry with Aβ 1–40 antibody shows intense labelling of Aβ within cerebral vessels and plaque cores (**a**). A serial section labelled with the Aβ 1–42 antibody showing intense labelling of a large diffuse Aβ deposit (**b**). Phospho-tau positivity (*brown*) in neurites around Aβ (*red*) in a cored plaque and dyshoric vessel with CAA in hGH control11 (**c**, **d**). The ubiquitin antibody labels extensive neurites around a cored Aβ plaque in hGH-iCJD (**e**). Reactive astrocytes around a cored Aβ plaque revealed on double labelling for Aβ (6F/3D antibody-red) and GFAP (GFAP antibody-brown) in hHG-iCJD (**f**). Astrocytes (*brown*) surround a cored Aβ plaque (*red*) (**g**) and microglial cells (*brown*) are present within a cored Aβ plaque (*red*) in hGH control10 (**h**). Diffuse Aβ deposits in the parietal cortex in vCJD27 (**i**). The diffuse cortical Aβ deposits in vCJD 37 (*red*) are shown not to co-localise with the abundant PrP deposits (*brown*) (**j**). Patchy localised meningeal CAA in the occipital region in sCJD14 (**k**). APOE-4 positivity in diffuse Aβ deposits in hGH control6 (**l**). The *bar* in **a** represents 100 µm for **a**–**b**; 20 µm for **f**, **h**; 25 µm for **c**–**e**, **g**, **j**, **l**; and 50 µm for **i**, **k**

Similar patterns of CNS Aβ accumulation were identified in five of the 12 hGH control cases examined. Three of these five Aβ positive cases contained parenchymal Aβ deposits only with a single case containing meningeal and intraparenchymal CAA, but no parenchymal deposits or capillary CAA (Figs. 4, 5i, j). The remaining case (hGH-control11) had extensive meningeal, intraparenchymal and capillary CAA widespread diffuse and neuritic cerebral cortical plaques with scanty neurites (up to CERAD score 2), and patchy subpial Aβ deposits (Fig. 5k, l). Dyshoric CAA vessels were also present in this case and occasional Aβ-laden vessels exhibited splitting of the vessel wall (Fig. 5m–p). Immunohistochemistry for Aβ 1–40 and Aβ 1–42 showed similar results for hGH-iCJD and hGH control cases with preferential labelling of CAA and plaque cores with Aβ 1–40, while the Aβ 1–42 antibody showed more labelling of diffuse Aβ deposits in the subpial region, diffuse plaques and the diffuse corona around some cored plaques (Fig. 6a, b). Sparse phospho-tau positive neurites were identified around cored Aβ plaques and around some large Aβ deposits surrounding intraparenchymal blood vessels in the hGH control cases (Fig. 6c, d). Phospho-tau-positive neurites were also sparse in hGH-iCJD cases, but more neurites were identified following labelling with the ubiquitin antibody (Fig. 6e). Reactive astrocytes and microglia were identified around and within cored Aβ plaques in both hGH-iCJD and hGH control cases (Fig. 6f–h).

In contrast to hGH-iCJD cases, the diffuse Aβ plaques in the occipital and parietal cortex of two vCJD cases (vCJD22 and vCJD32) appeared to co-localise with the PrP amyloid in some florid plaques. However, double IHC with antibodies to Aβ and PrP showed separation of some diffuse Aβ deposits from the florid plaques and other forms of focal PrP accumulation in the cortical grey matter in vCJD (Fig. 6i, j). No cored or neuritic Aβ plaques were identified and no meningeal or parenchymal CAA was present. A single sCJD control case (sCJD14) showed focal Aβ deposits in the walls of occasional small- to medium-sized meningeal blood vessels over the occipital and temporal lobes and in the superficial occipital cortex, with very few vessels exhibiting circumferential deposition (Fig. 6k). No PrP labelling in these vessels was identified.

The ABC and CAA scores for the hGH-iCJD, hGH control, vCJD and sCJD cases are summarised in Table 1.

No Aβ labelling was identified in either the CNS parenchyma or blood vessels in three of the hDM cases with sufficient paraffin tissue available further pathological analysis by immunohistochemistry.

### Phospho-tau, α-synuclein and TDP-43 pathology

Phospho-tau immunolabelling was present in all CJD cases analysed, irrespective of aetiology in the form of small neuritic dots in the neuropil as previously described [36]. Labelling of fine neuritic processes was noted around kuru plaques in sCJD controls and florid plaques in vCJD controls (Online Resource Fig. 1). Small numbers of AT8-positive pretangles labelled in the superior frontal cortex in a single hGH-iCJD (hGH-iCJD31) patient with idiopathic hGH deficiency. No Aβ or other adjacent pathology was observed and no phospho-tau positivity was identified in any other brain region. Small numbers of AT8-positive pretangles and occasional neurofibrillary tangles labelled in the gliotic region in the inferior right temporal cortex in a single hGH control patient who had undergone resection of an ependymoma (Online Resource Fig. 1). This localised abnormality was not associated with Aβ deposition. No α-synuclein or TDP-43 labelling was found in any of the cases examined.

### Correlations with Aβ pathology in hGH-treated patients

**Fig. 7:**
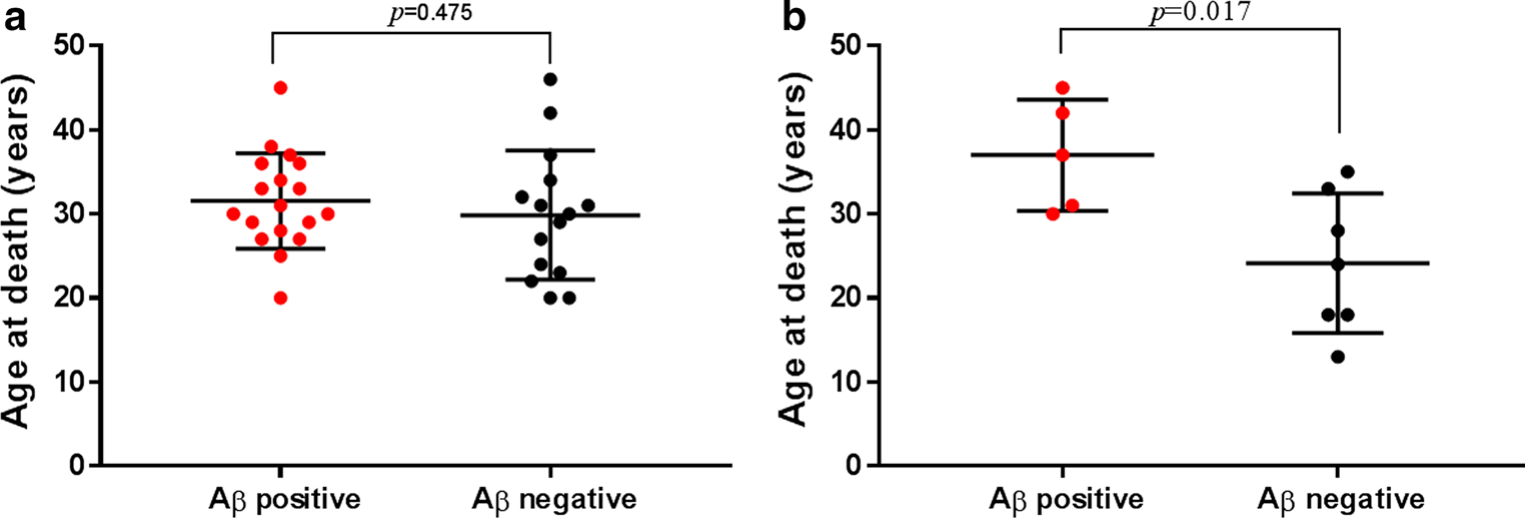
CNS accumulation and age at death in hGH-iCJD and hGH control cases. Comparisons of the age at death for **a** hGH-iCJD and **b** hGH-control patients in relation to accumulation of Aβ. No significant difference in the age at death was found between the Aβ-positive and Aβ-negative hGH-iCJD cases. However, a significant difference was found between the Aβ-positive and Aβ-negative hGH control cases, with the Aβ-positive cases showing a higher age at death. Statistical analysis was performed using an unpaired *t* test

**Fig. 8:**
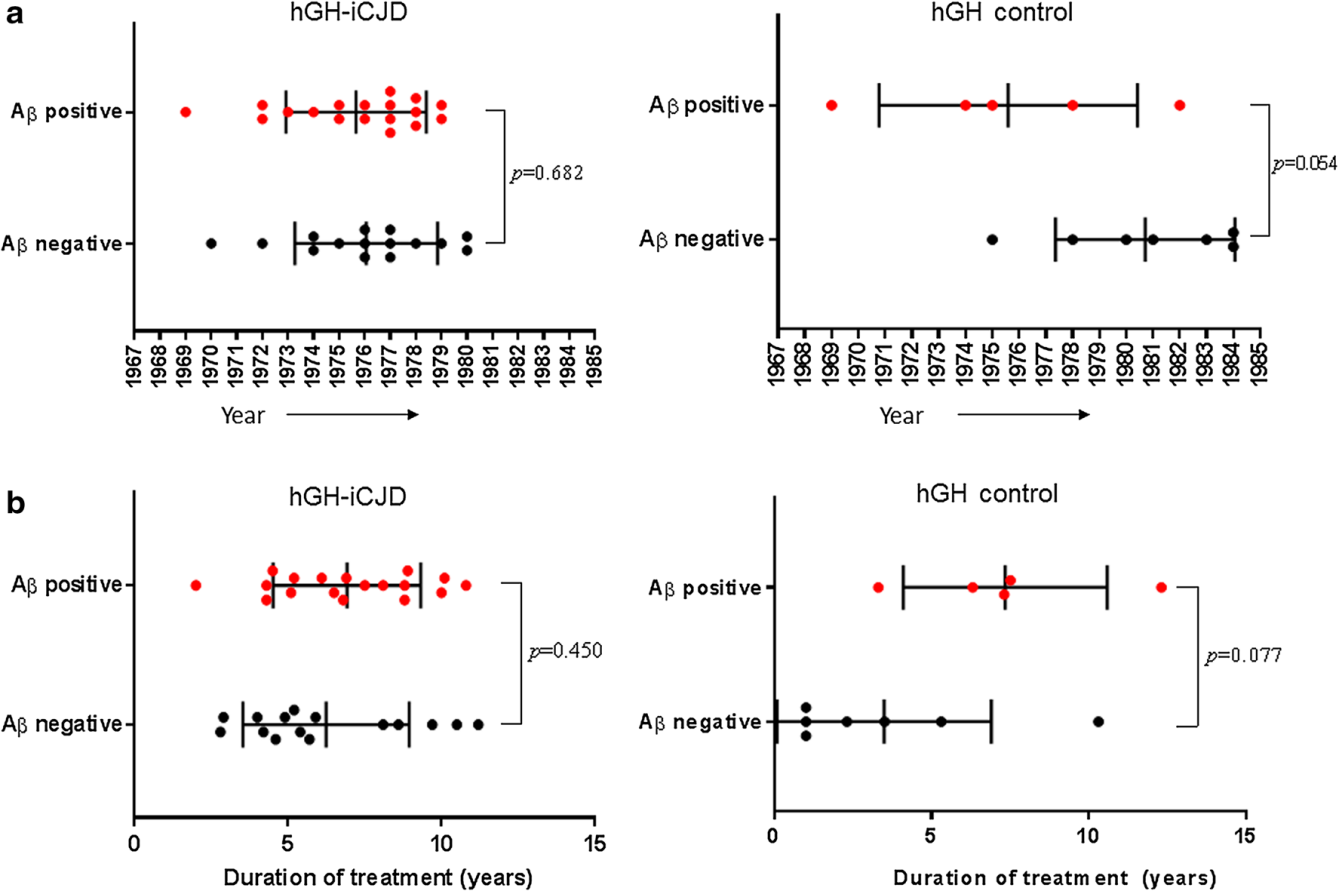
Treatment times with hGH-iCJD and hGH control patients. **a** Shows the year of first treatment for hGH-iCJD and hGH-control patients in relation to accumulation of Aβ and **b** shows duration of treatment for hGH-iCJD and hGH-control patients in relation to CNS Aβ accumulation. Differences were found in both the year of first hGH treatment and duration of treatment in the hGH control cases with and without CNS Aβ accumulation, but these did not reach levels of statistical significance. Statistical analysis was performed using an unpaired *t* test

The frequency of Aβ-positive cases in both the hGH-iCJD and hGH control groups are significantly higher than in the sCJD and vCJD age-matched controls (Fig. 3). No predisposing risk factors for Aβ deposition was identified in the clinical histories available for the hGH-treated patients. Analysis of the age range at death found no statistical difference when comparing the 18 Aβ-positive hGH-iCJD (20–45 years) cases with the 15 Aβ-negative hGH-iCJD (20–46 years) cases. However, statistically significant differences were found when comparing the five Aβ-positive (30–45 years) and seven Aβ-negative (13–35 years) hGH control cases (Fig. 7). No significant difference was observed in the date of first treatment or duration of hGH treatment when comparing the Aβ-positive hGH-iCJD and Aβ-negative hGH-iCJD cases, but there were non-significant differences suggesting that the Aβ-positive hGH control were treated earlier and for longer than the Aβ-negative hGH control cases (Fig. 8). No significant differences were found in either disease incubation period or duration of illness in relation to Aβ accumulation in the hGH-iCJD patients (Online Resource Fig. 2). The four hGH recipients who did not receive hGH produced by the modified Wilhelmi method had no evidence of Aβ accumulation in the CNS and did not develop iCJD (Online resource Table 2).

### *APOE* genotype and apoE-4 phenotype analysis

*APOE* genotype data were available for 22/33 hGH-iCJD cases and 2/12 hGH control cases analysed for Aβ (Online Resource Table 2; Table 2). No *APOE*-ɛ4 homozygous genotypes were found and a mixture of *APOE*-ɛ2/3, *APOE*-ɛ3/3 and *APOE*-ɛ3/4 genotypes was present in the hGH-iCJD cases. Immunohistochemistry for apoE-4 in five Aβ-positive hGH-iCJD cases found no positives (Table 2). In the five hGH control cases with CNS Aβ accumulation there was a single case with the *APOE*-ɛ3/3 genotype and another with the *APOE*-ɛ3/4 genotype. Immunohistochemistry for apoE-4 in the three remaining Aβ-positive hGH control cases found a single positive case (hGH control 6) (Fig. 6l; Table 2). As previously reported [60], only the parenchymal Aβ deposits and occasional neurites were labelled with the antibodies to apoE-4. Consequently, no information on the *APOE* genotype or apoE-4 phenotype can be obtained on the remaining 6 hGH-iCJD cases and the 7 hGH control cases with no CNS Aβ accumulation, due to a lack of frozen tissue samples and the absence of Aβ pathology. Comparison of *APOE* genotypes and Aβ accumulation found no statistical differences between the Aβ-positive and Aβ-negative hGH-iCJD cases; comparisons between the Aβ-positive and Aβ-negative hGH control cases was hampered by a lack of *APOE* genotype data for the Aβ-negative cases (Online Resource Fig. 3). The two vCJD cases with diffuse Aβ parenchymal deposits included one case with the *APOE*-ɛ3/4 genotype and one case with the *APOE*-ɛ2/3 genotype.

### Exome sequencing

Exome sequence analysis data for the hGH-iCJD cases, hGH-controls and sCJD cases with Aβ accumulation and with frozen tissue available found no coding, non-coding or 3′ or 5′ untranslated variants in genes that were likely to be associated with Aβ accumulation in the CNS. However, one of the vCJD cases (vCJD27) with diffuse Aβ deposits in the brain had the *PSEN1* p.E318G variant that increases the risk of AD in *APOE-ɛ*4 carriers [2] and a −48 C/T polymorphism in the *PSEN1* promoter that is a genotype associated with an increased risk of AD and an increased Aβ load in the brain [37]. Neither can be considered as highly penetrant monogenic alleles causing disease. No other variants were detected in the vCJD cases.

### hGH-iCJD and hGH control non-CNS tissues

**Fig. 9:**
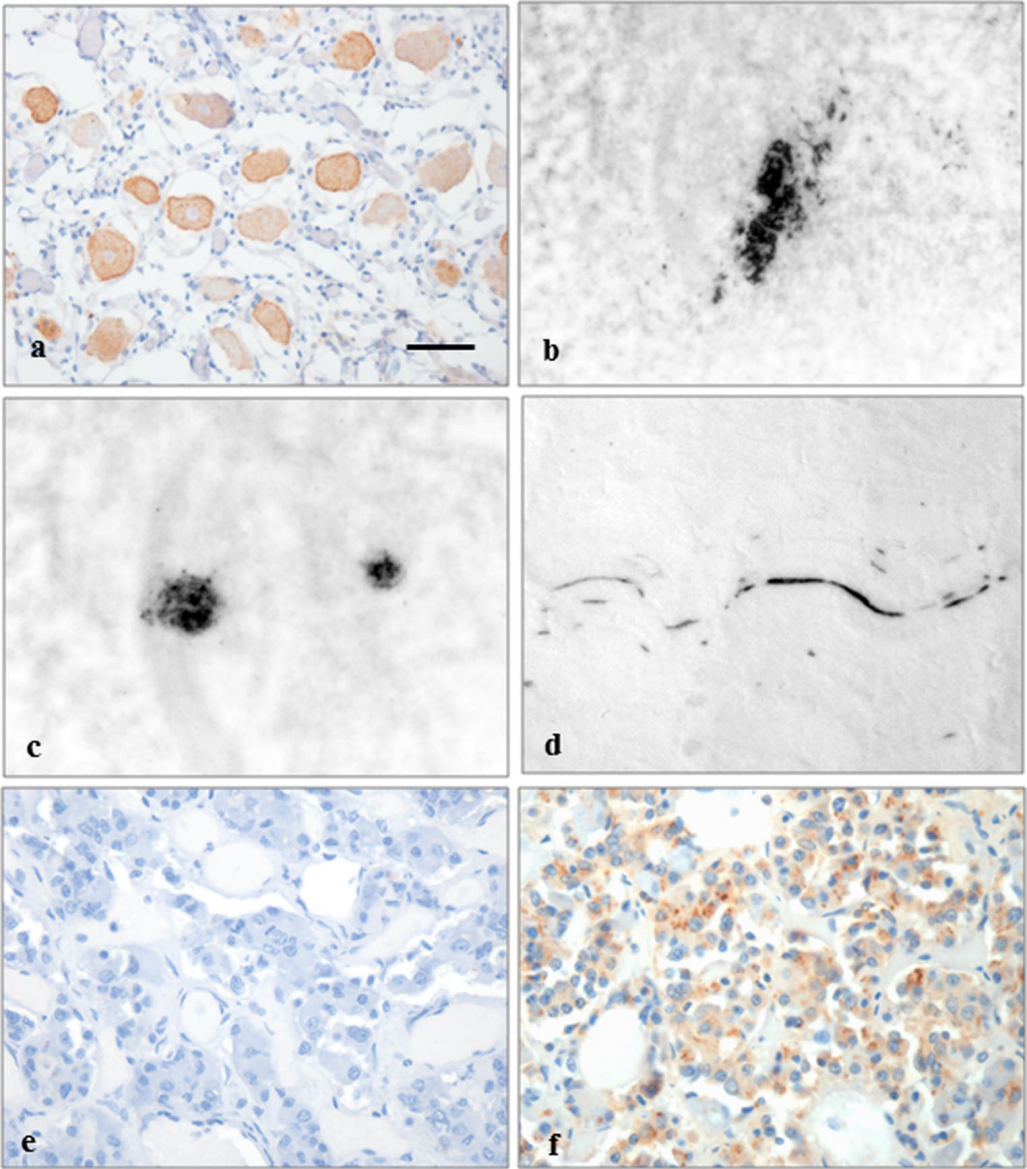
PrP accumulation in non-CNS tissues in hGH-iCJD cases. **a** Immunohistochemistry for the prion protein shows labelling of the ganglion cells in a dorsal root ganglion. **b** PET blot analysis with the 12F10 anti-PrP antibody shows intense labelling (*black*) of a group of chromaffin cells within the adrenal medulla, **c** in germinal centres within an abdominal lymph node and **d** in a small nerve within skeletal muscle. **e** Immunolabelling for Aβ using the 6F/3D antibody shows no labelling in the anterior pituitary gland, but **f** the 4G8 anti-Aβ antibody shows some diffuse fine granular and discrete dot-like intracellular positivity in the endocrine cells. The *bar* in **a** represents 50 µm for **a**, **b**, **d**; 25 µm for **e**, **f**; and 75 µm for **c**

Twenty-one different non-CNS tissues from 19 hGH-iCJD and two hGH controls were available for analysis. There was considerable variability in the availability of fixed and frozen tissue for each individual case. Details of the non-CNS tissues analysed and results of the analysis are summarised in Online Resource Table 4. Immunohistochemistry and PET blotting found accumulation of PrP in ganglion cells in the trigeminal ganglia and dorsal root ganglia (DRG) in all hGH-iCJD cases investigated (Fig. 9a). PrP immunopositivity was found in the adrenal gland in three hGH-iCJD cases, generally in the chromaffin cells of the adrenal medulla (Fig. 9b). In addition, PrP accumulation was observed in the germinal centres in an abdominal lymph node in a single hGH-iCJD case (hGH-iCJD15) (Fig. 9c). PrP immunoreactivity was also observed in a single skeletal muscle sample (hGH-iCJD15), in a linear pattern with a distribution and morphology consistent with that of a small nerve, as previously reported (Fig. 9d) [51]. Over half of the pituitary glands examined showed PrP immunoreactivity predominantly in the neurohypophysis, as previously described (Fig. 9e, f) [52]. No PrP immunoreactivity was observed in the appendix, duodenum, large intestine, peripheral nerve, small intestine, spleen, sympathetic chain or tonsil in the hGH-iCJD patients or in the hGH control tissues by either IHC or PET blot analysis.

Frozen, non-CNS tissue samples were available for analysis in 11 of the 35 hGH-iCJD cases. Western blot analysis following NaPTA precipitation (NaPTA WB) found unequivocal positive reactions in a single DRG and the pituitary glands in three cases. Two of the pituitary glands were found to have a type 2 PrP^res^ isoform (with a type 2 and type *i* + 2 in the corresponding brain samples), while the remaining case had a type 1 isoform (Online Resource Fig. 4). The brain in the latter case was found to have a type 2 PrP^res^ isoform in diagnostic Western blotting. Densitometric analysis indicated that the levels of PrP^res^ in the pituitary gland and dorsal root ganglion were around 0.8 and 0.04%, respectively, in comparison with brain PrP^res^ levels in a control hGH-iCJD case. Indeterminate results on NaPTA WB (when two of the usual three PrP^res^ bands were visible), was observed with adrenal gland, bone marrow, kidney, heart, lymph node, skeletal muscle and peripheral nerve. Skeletal muscle tissue from one case was previously found to be positive on NaPTA WB [51]. Repeat NaPTA WB analysis of the indeterminate cases was attempted, but was restricted in some cases by tissue sample availability. None of the repeat NaPTA WB tests altered the classification of these samples. Immunohistochemistry for Aβ showed no labelling in any of the non-CNS tissues in either the hGH-iCJD and hGH control cases.

## Discussion

This study provides a detailed description of the pathological phenotype of the largest series of iCJD cases (35 cases) occurring in recipients of hGH in the UK (or indeed any country). In doing so, we extend our earlier findings on the neuropathological phenotype of 21 (with frozen tissue) of these 35 cases, described as part of a thorough molecular and genetic analysis of UK hGH-iCJD cases [57]. Perhaps more importantly, it also provides a thorough investigation of the presence of Aβ, phospho-tau, α-synuclein and TDP-43 in the hGH-iCJD cases and in non-iCJD hGH recipients to establish whether there is evidence of iatrogenic seeding of these disease-associated proteins and whether this is independent of CJD transmission [28, 29].

### Neuropathological phenotypes of UK hGH-iCJD patients and comparison with sCJD

Iatrogenic CJD in hGH recipients is thought to result from contamination of hGH preparations with prions present in the pituitary gland collected from hGH extraction. PrP^res^ has been detected in the pituitary gland in sCJD and vCJD patients [52] and, as reported in this study, in the pituitary gland in patients with hGH-iCJD. The most likely source of prions in hGH preparations is sCJD, which is the commonest form of human prion disease, occurring most frequently in elderly patients, who accounted for the majority of the UK hospital autopsies that were the main source of pituitary glands collected for hGH extraction. The pathological phenotype in sCJD is determined largely by the *PRNP* codon 129 genotype of the patient and the PrP^res^ isoform in the brain [48]. The results from this study indicate that hGH-iCJD is subject to similar influences. The majority of the 35 hGH-iCJD cases showed a similar neuropathological phenotype to the recognised sCJD subtypes (Online Resource Table 1). All *PRNP* codon 129 VV UK hGH-iCJD show a pathological phenotype closely similar to the sCJD VV2 subtype. Of the 15 *PRNP* codon 129 MV hGH-iCJD cases, 14 show a pathological phenotype similar to the sCJD MV2K subtype, with the presence of kuru plaques. In contrast, the *PRNP* codon 129 MM subgroup show a divergent phenotype, with two of the four MM cases resembling the sCJD MM1 subtype in terms of neuropathology. The other two *PRNP* codon MM cases had kuru plaques and plaque-like deposits in the CNS. In our earlier publication, we suggest that this divergent neuropathological phenotype, combined with an intermediate (20 kDa) brain PrP^res^ isoform, may be a reflection of incomplete adaptation of the infecting V2 prion strain in the *PRNP* codon 129 MM host [57]. This pathology has not been described in UK cases of either hDM-iCJD or sCJD with a *PRNP* codon 129 MM genotype, but they have been described in cases of hDM-iCJD in Japan in *PRNP* codon 129 MM genotype patients. Kobayashi et al. [33] have suggested that the combination of a *PRNP* codon 129 genotype and the presence of kuru plaques in the cerebellum and cerebral cortex in a patient with apparently sporadic CJD should lead to a suspicion of an iatrogenic route of infection. In view of these results, particular care must be taken in interpreting the neuropathological findings in the five cases in which neither *PRNP* codon 129 genotype nor PrP^res^ isoform data are available (Online Resource Table 1). Case hGH-iCJD33 had a pathology that resembled the sCJD MV2K histotype; however, the possibility that this case could have a MM genotype cannot be excluded. Even if this were so, over 90% (32/35) of the UK hGH-iCJD cases would have neuropathological and biochemical features that are indistinguishable from one of the sCJD histotypes. With such close similarities in the neuropathological phenotypes between sCJD and hGH-iCJD cases, a detailed clinical history of any potential iatrogenic exposure to CJD prions is essential for diagnosis.

### Peripheral pathogenesis in hGH-iCJD

The peripheral pathogenesis of hGH-iCJD following inoculation of infected hGH preparations by intramuscular and subcutaneous injection is not known. Some prion strains (including vCJD) replicate in lymphoid tissues before invading the CNS. Neuroinvasion may occur via the blood-borne route, or by slow retrograde spread along autonomic nerve to the spinal cord and/or brainstem, then spreading to the brain itself [40]. These complex mechanisms partly explain the lengthy incubation period in prion diseases [32]. The results of this study on a limited range of non-CNS tissues available give some support for involvement of the peripheral nervous system (nerves, dorsal root ganglia and trigeminal ganglia) and, for the first time, involvement of lymphoid tissues in a single hGH-iCJD case. It may be that hGH-iCJD has some similarities to vCJD in terms of its peripheral pathogenesis, but the levels of PrP^res^ detected in lymphoid and peripheral nervous system structures appear lower and more restricted in distribution than in vCJD.

### CNS Aβ in hGH-iCJD and hGH control cases and associations with Alzheimer’s disease

The possibility of transmission of other neurotoxic proteins that accumulate in the pituitary gland was supported by recent evidence of Aβ seeding in the brains of 4/8 hGH recipients who died with iCJD [29]. However, all eight patients had clinical iCJD, raising the possibility that Aβ pathology resulted from cross-seeding, or was in some other way contingent on the iatrogenic transmission of CJD. Aβ seeding around PrP^Sc^ deposits has been reported in human prion disease, particularly in genetic prion diseases associated with the formation of PrP^Sc^ amyloid plaques [9, 27, 44]. In this study, examining a significantly larger number of hGH-iCJD cases (including cases from all *PRNP* codon 129 genotypes), CNS Aβ accumulation was identified in the cerebral cortex and/or meningeal and intraparenchymal blood vessels (including capillaries) in 18/33 hGH-iCJD. Crucially, our study reports Aβ accumulation in 5/12 hGH recipient who did not die with iCJD, indicating that the Aβ pathology found in hGH recipients is independent of the development of clinical CJD and the pathological changes that underlie it. Aβ pathology was similar in both hGH-iCJD and hGH control patients, occurring as parenchymal deposits (diffuse subpial deposits, diffuse plaques and cored/neuritic plaques) and as CAA, or both parenchymal and CAA. Overall, no major differences are observed in the nature or range of severity of the pathology between the hGH-iCJD and hGH control groups. The parenchymal Aβ deposits in both groups also had similar astrocytic and microglial reactions, which have not been reported in previous studies on CNS Aβ accumulation in cases of iCJD [16, 29, 36].

While the parenchymal Aβ deposits in the brains of the hGH recipients show similarities to the deposits found in Alzheimer’s disease, the distribution of the Aβ pathology does not appear to resemble the pattern that is characteristic of Alzheimer’s disease, but shows a distribution more similar to that previously described in hDM-iCJD [36]. Other distinctions between the Aβ-positive hGH recipients and Alzheimer’s disease patients include the notable absence of neurofibrillary tangles, a paucity of phospho-tau-positive neurites around neuritic plaques, the young age at death of the hGH recipients and the absence of a clinical history of slowly progressive cognitive impairment. All the hGH recipients with CNS Aβ accumulation in our study were under the age of 45 years at death, most of whom did not have the *APOE-*ɛ3/4 genotype or apoE-4 phenotype on immunohistochemistry. In a recent study of the brains of 154 individuals between the ages of 30 and 50 years [53], Aβ deposition was not identified in any individuals under the age of 40 years, but was present in the brains of 13 individuals aged between 40–49 years in the form of diffuse plaques throughout the cerebral cortex. None of the cases with Aβ positivity had clinical evidence of dementia or mild cognitive impairment. All individuals with Aβ positivity carried 1 or 2 *APOE* ɛ4 alleles; however, of the 28 individuals aged 40–50 years with the *APOE-*ɛ3/4 genotype, 10 (36%) had Aβ deposition in the brain, but 18 (64%) did not, indicating that the Aβ deposition in the brain before the age of 50 years may occur in only around 1/3 of non-demented individuals with the *APOE-*ɛ3/4 genotype [53].

Most of the cases in this study with CAA had the *APOE-*ɛ3/3 genotype, including the two cases with capillary CAA. The hGH-iCJD case with the greatest amount of Aβ positivity (hGH-iCJD18) had the *APOE-*ɛ3/3 genotype (combined ABC and CAA score 8), as did the hGH control case (hGH-control11) with the greatest amount of Aβ accumulation in the CNS (combined ABC and CAA score 11). The single sCJD case with CAA had the *APOE-*ɛ3/4 genotype, while the two vCJD cases with diffuse Aβ parenchymal deposits comprised one case with the *APOE-*ɛ3/4 genotype and one case with the *APOE-*ɛ2/3 genotype. The latter (vCJD 27) was identified on exome sequencing to have the *PSEN1* p.E318G variant that increases the risk of AD in *APOE-*ɛ4 carriers (but possibly not relevant in the *APOE* ɛ2/3 genotype) [2], and the −48 C/T polymorphism in the *PSEN1* promoter that is associated with an increased risk of AD and an increased Aβ load in the brain [37], which might be of relevance to the finding of sparse diffuse Aβ brain parenchymal deposits at 30 years of age. Overall, our results indicate no apparent influence of the *APOE-*ɛ3/4 genotype or the apoE-4 phenotype on the presence of either parenchymal or vascular Aβ accumulation in the groups of hGH-iCJD and hGH control patients, which include a patient as young as 20 years of age with CAA and an apoE-4-ve phenotype on immunohistochemistry.

### Factors influencing CNS Aβ accumulation in hGH recipients

In considering the accumulation of Aβ in the CNS in hGH recipients, the assumption is that the source of the Aβ originates from Aβ deposits in the pituitary glands collected for hGH extraction [28, 29]. However, our investigations on limited numbers of non-CNS tissues yielded no evidence in favour of the involvement of these non-CNS tissues in the spread of Aβ to the CNS. In this study, no significant differences were found between the Aβ-positive and Aβ-negative cases in hGH recipients in terms of the time period or duration of their hGH treatment, although there was a trend for the hGH controls with Aβ pathology to have been treated in an earlier time period and for longer than the Aβ negative cases. The most severe Aβ pathology, tended to occur in the patients who had survived for the longest after the end of their hGH treatment, perhaps reflecting slowly progressive Aβ seeding and propagation in the CNS prior to death. The lack of any relationship between the duration of hGH treatment and the development of Aβ pathology may be taken to indicate that the amount of Aβ contaminating the hGH inocula was variable and unpredictable. The same could be said for the lack of a relationship between the duration of hGH treatment and the development of iCJD; prion contamination of the hGH inocula also having been variable and unpredictable.

All hGH recipients who developed iCJD in the UK, were treated for at least 6 months between 1967 and 1980 with hGH produced by the modified Wilhelmi protocol [64]. This study has found that all the hGH recipients with CNS Aβ accumulation had also been treated (for varying periods of time) with this same preparation. While not all patients treated with this preparation developed either hGH-iCJD or CNS Aβ accumulation, it is important to note that the four patients who were never treated with this hGH preparation did not develop either iCJD or show CNS Aβ accumulation (Online Resource Table 2). Subsequent studies of the hGH produced by the modified Wilhelmi protocol were reported in 1982 [63]. Using polyacrylamide gel electrophoresis and amino acid analysis of the high molecular weight fraction of this preparation this study found “aggregated hGH as well as other material not separated from hGH by the purification procedure”. While it is highly likely that this “other material” included PrP^Sc^, it may also have included Aβ aggregates that were neither sufficiently removed nor denatured by the Wilhelmi protocol for them to lose their capacity to act as a propagon [13].

Questions have been raised on the clinical background of the patients included in the study by Jaunmuktane et al., suggesting that the pre-existing and underlying conditions causing hGH deficiency in this patient cohort “could by themselves lead to Aβ pathology and abnormal brain structure” [15]. In the 33 patients analysed for Aβ deposits in this study, we found no clinical history or neuropathological evidence of traumatic brain injury as the cause of the hGH deficiency. Of the 13 hGH recipients who had brain tumours, three had received post-operative radiotherapy (Online Resource Table 2), none of whom had Aβ deposition in the CNS. In addition, no evidence of the more generalised disorders that can be associated with Aβ deposition in the CNS (as suggested by Feeney et al. [15]) such as epilepsy, fragile X syndrome, Down’s syndrome or Parkinson’s disease were found (Online Resource Table 2). Furthermore, the morphology and distribution of Aβ lesions and relative lack of phospho-tau pathology argue against an underlying traumatic aetiology for the Aβ pathology reported in this study. In the two cases with incidental focal isolated phospho-tau pathology (pretangles and occasional tangles) unrelated to areas of Aβ deposition, the localised abnormalities did not match the recent proposed diagnostic criteria for chronic traumatic encephalopathy [42] and did not resemble the early stages of tauopathies such as corticobasal degeneration [67]. In hGH control10, the accompanying neuronal loss and gliosis indicates longstanding focal brain tissue damage that may relate to previous neurosurgery. The pretangles in hGH-iCJD 31 are more difficult to explain, but might represent a local reaction to a previous focal insult no longer apparent in the post-mortem brain. Pretangles in the brains of very young individuals were identified in a large study by Braak et al. [7] in subcortical sites, but not in the cerebral cortex. However, subcortical pretangles or tangles were not found in this or any other case examined.

Aβ accumulation in the CNS has been reported in both hGH-iCJD cases [29] and hDM-iCJD cases [16, 22, 36]. However, the level of detail in these reports, varies in both the descriptive pathology of the CNS and the results of associated genetic investigations. This study adds considerably to these reports in terms of the number of cases studied, the inclusion of hGH control cases without iCJD, and in the young age of the patients included. The detailed neuropathological description provided is comparable with that in Kovacs et al. [36], particularly in relation to the nature and localisation of the Aβ deposition and its relationship to Alzheimer’s disease, but still falls short of resembling a full AD neuropathological phenotype. Table 3 summarises the key findings in these reports and compares their findings with the findings in this study.

**Table 3:** Comparison of results with other studies on CNS Aβ accumulation in iCJD (modified from Kovacs [35])

Study references	Jaunmuktane et al. [29]	Frontzek et al. [16]	Kovacs et al. [36]	Hamaguchi et al. [22]	This study	
Clinical phenotype	CJD	CJD	CJD	CJD	CJD	Not CJD
Cause of iCJD	hGH	hDM	hDM	hDM	hGH	None
Number of cases	8	7	2^a^	16	33^b^	12
Other autopsy tissues examined	No	No	Yes	No	Yes	Yes
Non-CJD autopsy tissues examined	Pituitary gland: 55 cases	No	Dura mater: 84 cases	No	hGH control cases—see Online Resources Table 4	
Genetic analysis	*APOE* + AD genes	No	*APOE* + AD genes	*APOE*	*APOE* + AD genes	
Number with Aβ parenchymal deposits	4 + 2 focal	5	2^a^	13	12	4
Morphology and distribution of Aβ deposits described	Yes, with quantitative analysis	No	Yes, in detail	Subpial Aβ accumulation plaque morphology not included	Yes, in detail	
Age of cases with parenchymal Aβ	5th decade—51 years	28–63	28, 33	35–81	27–45	30–45
Age of cases <40 years with parenchymal Aβ	36 (focal deposits)	28, 33	28, 33	35, 39	27–38	30–37
Number with Aβ CAA	3 + 1 focal	5	2^a^	11	14	2
Age of cases with Aβ CAA	5th decade—51 years	28–63	28, 33	35–81	20–37	42–45
Age of cases <40 years with Aβ CAA	None	28, 33	28–33	35–39	20–37	None
AD-related phospho-tau pathology	No	No	No	Yes—details given	Yes—details given	
Statistically significant difference from sCJD	Yes	Yes	Yes	Yes	Yes—and also from vCJD	

*Aβ* amyloid beta, *AD* Alzheimer’s disease, *APOE* apolipoprotein E gene, *CAA* cerebral amyloid angiopathy, *CJD* Creutzfeldt–Jakob disease, *hDM* human dura mater, *hGH* human growth hormone, *i* iatrogenic, *s* sporadic, *v* variant, *y* years

^a^Cases also included in the study by Frontzek et al. [16]

^b^The total number of cases included in this study was 35, but two had insufficient paraffin-embedded tissue for further immunohistochemical analysis

## Conclusions and implications of results

This comprehensive study on the largest number of hGH-iCJD cases reported to date, indicates that Aβ can behave as a propagon in humans, able to spread to the CNS following intramuscular or subcutaneous injection and subsequently seed in the parenchyma of the brain and in cerebral blood vessels, but does not result in clinical Alzheimer’s disease or any other apparent clinical manifestations in these patients. CNS Aβ accumulation occurred in around 50% of hGH-iCJD and hGH control cases, and is therefore not dependent on co-existing PrP^Sc^ accumulation or other CJD pathology. The proposed behaviour of Aβ as a propagon in humans has broader implications including potential exposure to Aβ, for example in the re-use of Aβ-contaminated neurosurgical instruments, previously used on the brains of elderly patients, or via blood transfusions from elderly donors who may have increased levels of plasma Aβ [3]. However, recent epidemiological studies have found no evidence of either previous surgery or blood transfusion as risk factors for Alzheimer’s disease [11, 68]. These findings might also be taken to indicate that a significant number of the remaining survivors in the cohort of UK hGH recipients are at increased risk of CNS Aβ accumulation and, although they may not progress to symptomatic Alzheimer’s disease, they may subsequently develop vascular complications. The severe CAA found in the older hGH control patients in this study suggests that surviving hGH recipients may be at future risk of the complications of CAA, including spontaneous lobar cerebral haemorrhage, perivascular inflammation and cognitive impairment [17] in addition to having lived with the knowledge of an increased risk of CJD.

## Electronic supplementary material

Below is the link to the electronic supplementary material.

Supplementary material 1 (DOCX 773 kb)
