# Supplementary material for: Amyloid-β accumulation in the CNS in human growth hormone recipients in the UK
Source: Acta Neuropathol. 2017 Mar 27;134(2):221–40. doi: 10.1007/s00401-017-1703-0 (PMC5508038; doi:10.1007/s00401-017-1703-0)
Supplement: Supplementary file 1 — Supplementary material 1 (DOCX 773 kb) [file 401_2017_1703_MOESM1_ESM.docx]

**Acta Neuropathologica Online Resource**

**Amyloid-β accumulation in the CNS in human growth hormone recipients in the UK**

Diane L. Ritchie^1^, Peter Adlard^2^, Alexander H. Peden^1^, Suzanne Lowrie^1^, Margaret Le Grice^1^, Kimberley Burns^1^, Rosemary J Jackson^3^, Helen Yull^1^, Michael J. Keogh^4^, Wei Wei^5^, Patrick F. Chinnery^5^, Mark W. Head^1^, James W. Ironside^1^

Author for correspondence: Dr Diane Ritchie, Bryan Matthews Building, Western General Hospital, Edinburgh, EH4 2XU, United Kingdom. Tel: +44 (0)131 537 2378, Fax: +44 (0)131 343 1404, E-mail: [diane.ritchie@ed.ac.uk](mailto:diane.ritchie@ed.ac.uk)

**Figures**

**Figure. 1**

**
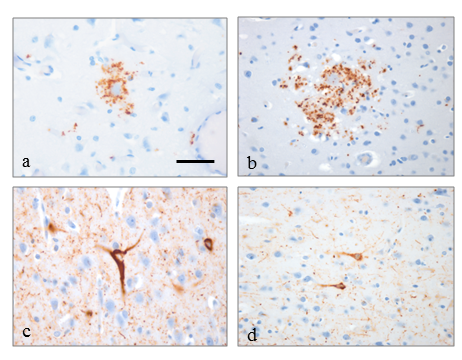
**

**Figure 1: Non-Aβ related tau pathology in vCJD, hGH-iCJD and hGH control cases.** Small neurites around a kuru-type plaque in hGH-iCJD (a). Numerous small rounded neurites and threads are present around a florid plaque in vCJD (b). Occasional neurofibrillary tangles and numerous neuropil threads are present in the focally gliotic temporal cortex in hGH control10 (c). Occasional pretangles and neuropil threads are present in a small region within the superior frontal cortex in hGH-iCJD31. No glial positivity was identified (e). All sections were stained with the AT8 anti-phosphotau antibody. The *bar* in image “a” represents 20µm for a-c; and 25µm for d.

**Figure. 2**

**
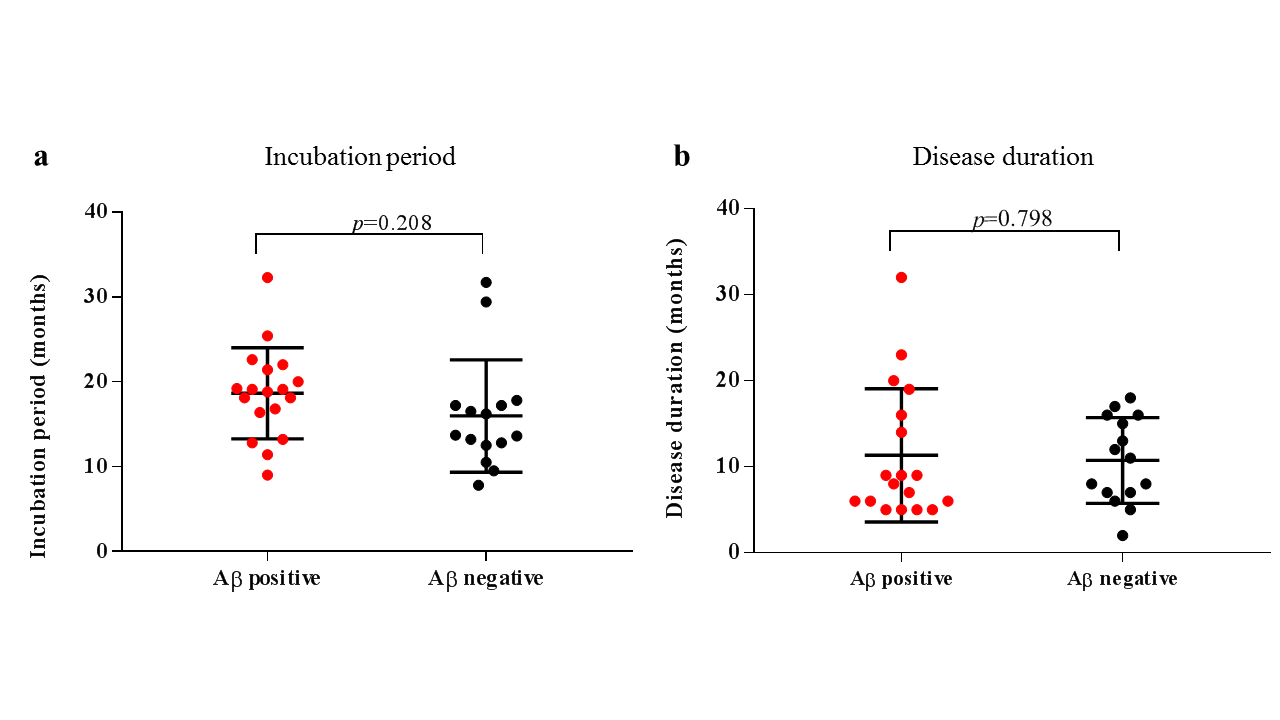
**

**Figure 2: Disease incubation period and duration of illness in hGH-iCJD cases in relation to CNS Aβ positivity**. (a) Incubation periods and (b) durations of disease for hGH-iCJD in relation to CNS Aβ accumulation. Differences were found in the incubation period and the disease duration between the Aβ positive and Aβ negative hGH-iCJD groups, but these did not reach statistical significance. Horizontal bars represent mean with standard deviation values. Statistical analysis was performed using an unpaired t-test.

**Figure. 3**

**
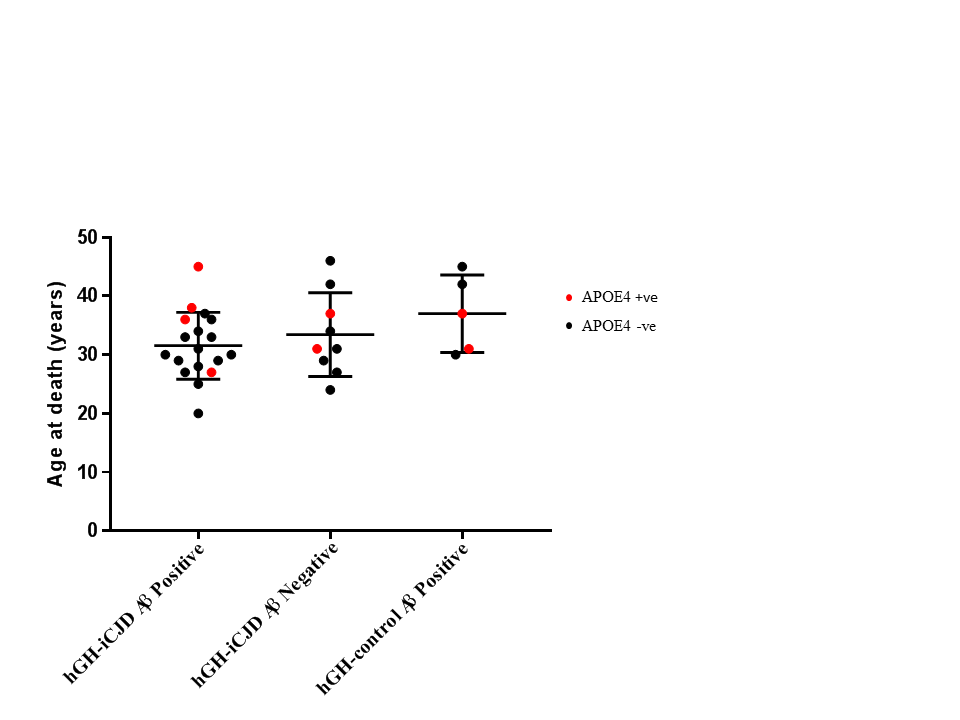
**

**Figure 3: Distribution of *APOE* ɛ3/4 genotypes and apoE-4 positive phenotypes in relation to age at death and CNS Aβ accumulation in hGH-iCJD and hGH control cases**. No apparent relationship between age and APOE4 status is detected. There is no evidence of a difference in the percentage of APOE4+ve hGH-iCJD cases in the Aβ positive and Aβ negative groups; p=1.00 (Fisher’s exact test). There is no evidence of a significant difference in the percentage of APOE4+ve cases between the Aβ positive hGH-iCJD group and the Aβ positive hGH control group; p=0.576 (Fisher’s exact test). Horizontal bars represent mean with standard deviation values.


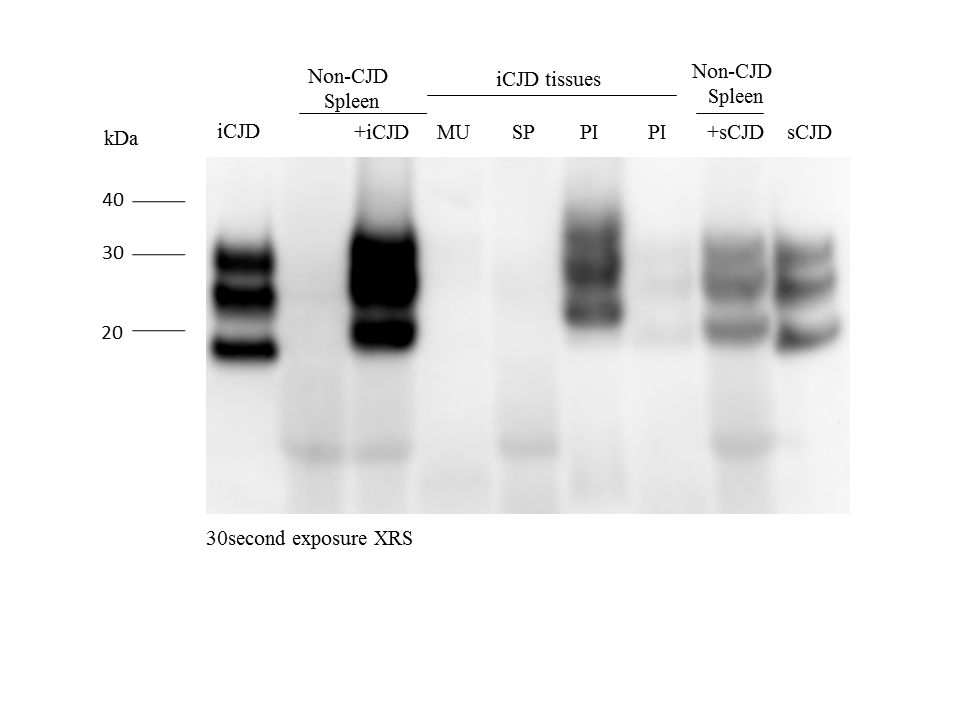
**Figure. 4**

**Figure 4:** **Western blots with NaPTA precipitation on non-CNS tissues**. Western blot analysis after NaPTA precipitation showing (left to right) iCJD brain, non-CJD spleen, non-CJD spleen spiked with iCJD brain, iCJD muscle, iCJD spleen, iCJD pituitary 1, iCJD pituitary 2, non-CJD spleen spiked with sCJD brain, sCJD brain. iCJD pituitary 1 gives a strong positive signal, with a weaker signal for iCJD pituitary 2 (confirmed on a longer exposure). No signal is seen in the lanes for iCJD muscle and spleen. kDa,kilodaltons; MU, muscle; PI, pituitary; SP, spleen

**Tables**

**Table 1: Clinical and genetic features of patients examined**

| Study ID | Sex | Age at death (years) | Age at disease onset in years (year of onset) | Incubation period (years) | Duration of illness (months) | *PRNP* codon 129 genotype | PrP^res^ type† | Histotype |
| --- | --- | --- | --- | --- | --- | --- | --- | --- |
| hGH-iCJD1***** | m | 34 | 33 (1989) | 17 | 8 | MV | i+2 | MV2K+2C |
| hGH-iCJD2 | m | 31 | 30 (1991) | 17 | 6 | VV | 2 | VV2 |
| hGH-iCJD3 | m | 27 | 26 (1992) | 14 | 17 | MV | i+2 | MV2K |
| hGH-iCJD4 | m | 30 | 29 (1992) | 13 | 9 | VV | 2 | VV2 |
| hGH-iCJD5 | m | 25 | 25 (1993) | 11 | 5 | VV | 2 | VV2 |
| hGH-iCJD6 | f | 33 | 32 (1994) | 18 | 6 | VV | 2 | VV2 |
| hGH-iCJD7 | f | 31 | 30 (1995) | 16 | 12 | MV | i+2 | MV2K+2C |
| hGH-iCJD8 | m | 29 | 28 (1996) | 17 | 6 | VV | 2 | VV2 |
| hGH-iCJD9 | m | 33 | 32 (1985) | 18 | 14 | MV | i+2 | MV2K |
| hGH-iCJD10 | m | 36 | 36 (1996) | 19 | 8 | VV | 2 | VV2 |
| hGH-iCJD11 | m | 37 | 35 (1996) | 18 | 18 | MV | i+2 | MV2K +2C |
| hGH-iCJD12 | m | 27 | 27 (1998) | 16 | 5 | VV | 2 | VV2 |
| hGH-iCJD13 | m | 34 | 32 (1997) | 17 | 16 | MV | i+2 | MV2K |
| hGH-iCJD14 | m | 29 | 28 (1997) | 16 | 15 | MV | i+2 | MV2K |
| hGH-iCJD15 | m | 37 | 37 (1999) | 20 | 9 | MV | i+2 | MV2K |
| hGH-iCJD16 | m | 30 | 28 (2000) | 20 | 16 | MV | i+2 | MV2K |
| hGH-iCJD17***** | f | 41 | 38 (2001) | 23 | 22 | MV | i+2 | MV2K |
| hGH-iCJD18 | f | 34 | 32 (2003) | 22 | 23 | MV | i+2 | MV2K |
| hGH-iCJD19 | f | 29 | 27 (2004) | 21 | 32 | MV | i+2 | MV2K |
| hGH-iCJD20 | m | 46 | 46 (2011) | 29 | 5 | MM | i | Atypical – resembles MV2K+2C |
| hGH-iCJD21 | m | 42 | 42 (2012) | 31 | 8 | MM | 1 | MM1 |
| hGH-iCJD22 | m | 20 | 19 (1997) | 8 | 7 | VV | N/A | Resembles  VV2 |
| hGH-iCJD23 | f | 20 | 20 (1990) | 10 | 2 | VV | N/A | Resembles  VV2 |
| hGH-iCJD24 | m | 32 | 31 (1992) | 14 | 13 | MV | N/A | ResemblesMV2K |
| hGH-iCJD25 | m | 30 | 29 (1991) | 13 | 16 | N/A | N/A | Resembles MM/MV1 |
| hGH-iCJD26 | m | 27 | 26 (1994) | 13 | 7 | VV | N/A | Resembles  VV2 |
| hGH-iCJD27 | m | 24 | 23 (1994) | 13 | 7 | VV | N/A | Resembles VV2 |
| hGH-iCJD28 | f | 22 | 22 (1984) | 10 | 11 | MV | N/A | Resembles MV2C |
| hGH-iCJD29 | m | 20 | 20 (1989) | 9 | 5 | N/A | N/A | Resembles VV2 |
| hGH-iCJD30 | m | 23 | 23 (1996) | 14 | 8 | N/A | N/A | Resembles VV2 |
| hGH-iCJD31 | m | 28 | 28 (1997) | 19 | 5 | MM | N/A | Atypical: some VV2- like features |
| hGH-iCJD32 | m | 36 | 35 (1999) | 21 | 9 | N/A | N/A | Resembles VV2 |
| hGH-iCJD33 | m | 31 | 29 (2000) | 18 | 20 | N/A | N/A | ResemblesMV2K |
| hGH-iCJD34 | m | 38 | 37 (2003) | 25 | 19 | MV | N/A | MV2K+2C |
| hGH-iCJD35 | f | 45 | 45 (2006) | 32 | 6 | MM | N/A | MM1 |
| hGH control1 | m | 18 | N/A | N/A | N/A | N/A | - | - |
| hGH control2 | f | 18 | N/A | N/A | N/A | N/A | - | - |
| hGH control3 | m | 13 | N/A | N/A | N/A | MV | - | - |
| hGH control4 | m | 28 | N/A | N/A | N/A | MV | - | - |
| hGH control5 | f | 24 | N/A | N/A | N/A | N/A | - | - |
| hGH control6 | f | 31 | N/A | N/A | N/A | N/A | - | - |
| hGH control7 | m | 30 | N/A | N/A | N/A | N/A | - | - |
| hGH control8 | m | 33 | N/A | N/A | N/A | MV | - | - |
| hGH control9 | m | 42 | N/A | N/A | N/A | N/A | - | - |
| hGH control10 | m | 37 | N/A | N/A | N/A | MV | - | - |
| hGH control11 | m | 45 | N/A | N/A | N/A | MM | - | - |
| hGH control12 | m | 35 | N/A | N/A | N/A | N/A | - | - |
| hDM-iCJD1* | f | 45 | 44(1990) | 8 (estimate) | 5 | MM | 1 | MM1 |
| hDM-iCJD2 | f | 27 | 24(1994) | 9 | 33 | MM | 1 | MM1 |
| hDM-iCJD3 | m | 34 | 33(2002) | 15 | 5 | MM | 1 | MM1 |
| hDM-iCJD4* | m | 31 | (1986) | 4 | 4 | N/A | N/A | Resembles VV2 |
| hDM-iCJD5 | m | 47 | (1992) | 7 | 11 | N/A | N/A | Resembles MM1 |

*****Insufficient paraffin-embedded tissue available for full pathological investigation

†PrP^res^ types assigned using the classification system used by Ritchie et al [57]

N/A – not available

**Table 2: Clinical data on hGH treated patients**

| Study ID | Duration of hGH treatment (years) | Midpoint of hGH treatment to death (years) | Duration of HWP treatment (years) | APOE genotype data | Original diagnosis |
| --- | --- | --- | --- | --- | --- |
| hGH-iCJD1***** | 4 | 17.5 | 2.5 | N/A | craniopharyngioma |
| hGH-iCJD2 | 10 | 16.9 | >4 | 3/3 | IGHD |
| hGH-iCJD3 | 6 | 14.9 | 3.8 | 2/3 | astrocytoma and radiotherapy |
| hGH-iCJD4 | 4 | 14.2 | 3.5 | 3/3 | IGHD |
| hGH-iCJD5 | 6 | 11.9 | 1.8 | 3/3 | IGHD |
| hGH-iCJD6 | 4 | 18.5 | 3 | 3/3 | IGHD |
| hGH-iCJD7 | 4 | 17.1 | 3.3 | 3/4 | IGHD |
| hGH-iCJD8 | 8 | 17.5 | 1 | 2/3 | IGHD |
| hGH-iCJD9 | 10 | 20.0 | 5.8 | 3/3 | IGHD |
| hGH-iCJD10 | 4 | 19.8 | 4.3 | 3/4 | IGHD |
| hGH-iCJD11 | 4 | 19.1 | 4 | 3/4 | IGHD |
| hGH-iCJD12 | 7 | 16.8 | 0.8 | 3/4 | IGHD |
| hGH-iCJD13 | 5 | 18.2 | 3.2 | 3/3 | IGHD |
| hGH-iCJD14 | 9 | 18.2 | 4 | 3/3 | histiocytosis X |
| hGH-iCJD15 | 6 | 20.4 | 3.8 | 3/3 | IGHD |
| hGH-iCJD16 | 3 | 21.5 | 1.8 | 3/3 | IGHD |
| hGH-iCJD17***** | 10 | 25.2 | 6 | N/A | IPHP |
| hGH-iCJD18 | 8 | 24.1 | 4.3 | 3/3 | IGHD |
| hGH-iCJD19 | 7 | 24.5 | 1.5 | 3/3 | IGHD |
| hGH-iCJD20 | 3 | 29.9 | 0.5 | 3/3 | IGHD |
| hGH-iCJD21 | 8 | 31.6 | 3.3 | 3/3 | craniopharyngioma |
| hGH-iCJD22 | 11 | 8.2 | 5.5 | N/A | IGHD |
| hGH-iCJD23 | 11 | 15.1 | 8.2 | N/A | IGHD |
| hGH-iCJD24 | 6 | 14.4 | 4.7 | N/A | IGHD |
| hGH-iCJD25 | 3 | 13.7 | 2.3 | N/A | IGHD |
| hGH-iCJD26 | 8 | 13.7 | 2 | E4-ve(IHC) | IGHD |
| hGH-iCJD27 | 6 | 13.1 | 1 | 3/3 | IGHD |
| hGH-iCJD28 | 5 | 10.4 | 1.5 | N/A | craniopharyngioma |
| hGH-iCJD29 | 9 | 9.3 | 3.8 | N/A | craniopharyngioma |
| hGH-iCJD30 | 5 | 14.2 | 1 | N/A | IGHD |
| hGH-iCJD31 | 2 | 19.7 | 2 | E4-ve(IHC) | IGHD |
| hGH-iCJD32 | 10 | 22.2 | 5 | E4-ve(IHC) | craniopharyngioma |
| hGH-iCJD33 | 7 | 19.8 | 3.8 | E4-ve(IHC) | IGHD |
| hGH-iCJD34 | 10 | 26.9 | >4.3 | 3/4 | IGHD |
| hGH-iCJD35 | 9 | 32.9 | 3.5 | 3/4 | Russell-Silver syndrome |
| hGH control1 | 1.0 | 2.7 | 0 | N/A | malignant glioma and radiotherapy |
| hGH control2 | 2.3 | 5.0 | 0 | N/A | craniopharyngioma |
| hGH control3 | 1.0 | 6.4 | 0 | N/A | craniopharyngioma |
| hGH control4 | 3.5 | 8.9 | 3 | N/A | craniopharyngioma |
| hGH control5 | 10.3 | 13.4 | 5.2 | N/A | craniopharyngioma |
| hGH control6 | 7.3 | 19.6 | 6 | E4+(IHC) | Prader-Willi syndrome |
| hGH control7 | 6.3 | 17.5 | 1.8 | E4+?(IHC) | craniopharyngioma |
| hGH control8 | 5.3 | 16.4 | 0.5 | N/A | mitochondrial cytopathy |
| hGH control9 | 7.5 | 21.6 | 3.2 | E4-(IHC) | IPHP |
| hGH control10 | 3.3 | 16.7 | 0.3 | 3/4 | Ependymoma |
| hGH control11 | 12.3 | 30.3 | 6.6 | 3/3 | IGHD |
| hGH control12 | 1.0 | 35.1 | 0 | N/A | IPHP |

*****Insufficient paraffin-embedded tissue available for full pathological investigation

IGHD - isolated growth hormone deficiency, IPHP: idiopathic panhypopituitarism

**Table 3: Antibodies and immunohistochemical protocols**

| **Antibody** | **Antigen Specificity** | **1^st^**  **Pre-treatment** | **2nd**  **Pre-treatment** | **3rd**  **Pre-treatment** | **Antibody dilution*/ Incubation time (mins) †** |
| --- | --- | --- | --- | --- | --- |
| 5A9 | Human apoE-4 protein | Autoclave 121^o^C 10mins | 96% FA  5 mins | - | 1:600/45 |
| 5B5 | Human apoE-4 protein | Autoclave 121^o^C 10mins | 96% FA  5 mins | - | 1:200/45 |
| D6E10 | Human apoE protein | Autoclave 121^o^C 10mins | 96% FA  5 mins | - | 1:2000/45 |
| 11A50-B10 | Human Aβ protein (1-40) | Autoclave 121^o^C 10mins | 96% FA  5 mins | - | 1:400/45 |
| 12F4 | Human Aβ protein (1-42) | Autoclave 121^o^C 10mins | 96% FA  5 mins | - | 1:3000/45 |
| 6F3D | Human Aβ  (residues 8-17 with additional C-terminal cysteine) | 96% FA  5 mins | - | - | 1:100/30 |
| 4G8 | Human Aβ  (Epitope 17-24) | 96% FA  5 mins | - | - | 1:45,000/30 |
| 5G4 | Human α-Synuclein | MW 10mM Citric Acid (pH6.0)  15 mins | 96% FA  1 min | - | 1:1200/30 |
| AT8 | Human PHF-Tau | MW 10mM Citric Acid (pH6.0)  15 mins | - | - | 1:500/30 |
| 11-9 | Human Phospho TDP-43 | MW 10mM Citric Acid (pH6.0)  15 mins | - | - | 1:4000/30 |
| 12F10 | Human prion protein  (Epitope 142-160) | Autoclave 121^o^C 10mins | 96% FA  5 mins | PK (5µg/ml)  5 mins | 1:1700/60 |
| KG9 | Human prion protein  (Epitope 140-180) | Autoclave 121^o^C 10mins | 96% FA  5 mins | PK (5µg/ml)  5 mins | 1:500/60 |
| PGM1 | CD 68 | MW 10mM Citric Acid (pH6.0)  15 mins | - | - | 1:100/30 |
| GFAP | Glial fibrillary acidic protein | - | - | - | 1:1000/30 |
| Ubiquitin | Ubiquitin | MW 10mM Citric Acid pH6.0,15 mins |  |  | 1:700/30 |
| 11-9 | Human phosphoTDP-43 (pS409/410) | MW 10mM Citric Acid (pH6.0)  15 mins |  |  | 1:4000/30 |

* All antibodies diluted in antibody diluent (Leica Biosystems, UK: Product RE7133-CE)

† All primary antibodies are incubated at room temperature

Aβ, amyloid beta; FA, formic acid, MW, microwave, PK, Proteinase K

**Table 4: Summary of PrP immunohistochemistry, PET blot and WB NaPTA results in non-CNS tissues in hGH-iCJD and control cases.**

|  | hGH-iCJD (n=19) | | | hGH-control (n=2) | | |
| --- | --- | --- | --- | --- | --- | --- |
|  | IHC | PET blot | NaPTA | IHC | PET blot | NaPTA |
| AD | 3/5 | 4/5 | 0/1 | 0/1 | 0/1 | 0/0 |
| AP | 0/4 | 0/4 | 0/1 | 0/2 | 0/2 | 0/0 |
| BM | 0/0 | 0/0 | 0/1 | 0/0 | 0/0 | 0/0 |
| DRG | 3/3 | 3/3 | 1/1 | 0/0 | 0/0 | 0/0 |
| DU | 0/1 | 0/1 | 0/0 | 0/0 | 0/0 | 0/0 |
| HE | 0/0 | 0/0 | 0/1 | 0/0 | 0/0 | 0/0 |
| KI | 0/0 | 0/0 | 0/2 | 0/0 | 0/0 | 0/0 |
| LI | 0/1 | 0/1 | 0/2 | 0/1 | 0/1 | 0/0 |
| LN | 1/6 | 1/6 | 0/3 | 0/1 | 0/1 | 0/0 |
| MU | 1/8 | 1/8 | 0/5 | 0/1 | 0/1 | 0/0 |
| PC | 0/0 | 0/0 | 0/2 | 0/0 | 0/0 | 0/0 |
| PI | 6/10 | 6/10 | 3/5 | 0/1 | 0/1 | 0/0 |
| PN | 0/7 | 0/7 | 0/3 | 0/0 | 0/0 | 0/0 |
| SG | 0/0 | 0/0 | 0/1 | 0/0 | 0/0 | 0/0 |
| SI | 0/1 | 0/1 | 0/1 | 0/0 | 0/0 | 0/0 |
| SP | 0/10 | 0/10 | 0/6 | 0/1 | 0/1 | 0/0 |
| SY | 0/2 | 0/2 | 0/1 | 0/0 | 0/0 | 0/0 |
| TG | 2/2 | 2/2 | 0/0 | 0/0 | 0/0 | 0/0 |
| TO | 0/3 | 0/3 | 0/1 | 0/2 | 0/2 | 0/0 |
| TY | 0/0 | 0/0 | 0/1 | 0/0 | 0/0 | 0/0 |

Abbreviations: AD: adrenal gland; AP: appendix; BM: bone marrow; DRG: dorsal root ganglion; DU: duodenum: HE: heart: KI: kidney; LI: large intestine; LN: lymph node; MU: muscle; PC: pancreas; PI: pituitary; PN: peripheral nerve; SG: salivary gland; SI: small intestine; SP: spleen; SY: sympathetic chain; TG: trigeminal ganglion; TO: tonsil; TY: thyroid.
